# The virulence factor regulator and quorum sensing regulate the type I-F CRISPR-Cas mediated horizontal gene transfer in *Pseudomonas aeruginosa*

**DOI:** 10.3389/fmicb.2022.987656

**Published:** 2022-09-30

**Authors:** Stephen Dela Ahator, Yang Liu, Jianhe Wang, Lian-Hui Zhang

**Affiliations:** ^1^Guangdong Province Key Laboratory of Microbial Signals and Disease Control, Integrative Microbiology Research Center, South China Agricultural University, Guangzhou, China; ^2^Research Group for Host Microbe Interactions, Department of Medical Biology, Faculty of Health Sciences, UiT The Arctic University of Norway, Tromsø, Norway; ^3^Centro de Biotecnología y Genómica de Plantas, Instituto Nacional de Investigación y Tecnología Agraria y Alimentaria (INIA), Universidad Politécnica de Madrid (UPM), Madrid, Spain

**Keywords:** CRISPR-Cas system, quorum sensing, horizontal gene transfer, virulence factor regulator, second messenger, CRISPR adaptation, calcium

## Abstract

*Pseudomonas aeruginosa* is capable of thriving in diverse environments due to its network of regulatory components for effective response to stress factors. The survival of the bacteria is also dependent on the ability to discriminate between the acquisition of beneficial and non-beneficial genetic materials *via* horizontal gene transfer (HGT). Thus, bacteria have evolved the CRISPR-Cas adaptive immune system for defense against the deleterious effect of phage infection and HGT. By using the transposon mutagenesis approach, we identified the virulence factor regulator (Vfr) as a key regulator of the type I-F CRISPR-Cas system in *P. aeruginosa*. We showed that Vfr influences the expression of the CRISPR-Cas system through two signaling pathways in response to changes in calcium levels. Under calcium-rich conditions, Vfr indirectly regulates the CRISPR-Cas system *via* modulation of the AHL-QS gene expression, which could be vital for defense against phage infection at high cell density. When encountering calcium deficiency, however, Vfr can directly regulate the CRISPR-Cas system *via* a cAMP-dependent pathway. Furthermore, we provide evidence that mutation of *vfr* reduces the CRISPR-Cas spacer acquisition and interference of HGT. The results from this study add to the regulatory network of factors controlling the CRISPR-Cas system in response to abiotic factors in the environment. The findings may facilitate the design of effective and reliable phage therapies against *P. aeruginosa* infections, as targeting Vfr could prevent the development of the CRISPR-Cas mediated phage resistance.

## Introduction

*Pseudomonas aeruginosa* is a ubiquitous opportunistic pathogen that thrives in diverse habitats and often infects immunocompromised patients, causing various forms of acute and chronic infections. *P. aeruginosa* is capable of transforming into a virulent pathogen upon sensing favorable changes in the host (Passador et al., 1993). This bacterium accounts for over 10% of nosocomial infections, making it a highly significant pathogen in hospital settings (Jones, 2010). It causes hard-to-treat infections due to the development of resistance mechanisms against most conventional antibiotics (Drenkard and Ausubel, 2002; Mah et al., 2003; Wolfgang et al., 2003a).

As *P. aeruginosa* becomes increasingly antibiotic-resistant, there is an urgent need for developing novel treatments and disease prevention strategies. Over the last few years, various non-antibiotic disease control treatments have been tested, including quorum quenching (Dong et al., 2007), bacterial vaccines, and phage therapy (Hoggarth et al., 2019). The interest in phage therapy is based on the ubiquity of bacteriophages (phages), host specificity, and their ability to cause detrimental effects on host cells reminiscent of the action of antibiotics. Phages attach to the bacterial host cell *via* surface receptors and inject their genetic material into the host cell. By hijacking the host’s molecular building blocks and enzymes, they replicate their genetic materials and produce more progeny phages that are released by the lysis of the host cell (Doss et al., 2017). To date, over 130 phages that attack *P. aeruginosa* have been reported with the fully sequenced genome (Hoggarth et al., 2019), signifying a large repository of natural genetic resources to be exploited in developing practical and effective phage therapy. However, similar to the development of antibiotic resistance, bacteria evolve resistance to phages, in part due to the clustered regularly interspaced short palindromic repeats (CRISPR) and the CRISPR Associated (Cas) proteins, which are widespread in bacteria and archaea (Makarova et al., 2015). This adaptive immune system is composed of a genomic CRISPR array with short sequences known as spacers acquired for previously encountered foreign genetic materials (Jansen et al., 2002). The acquisition of the spacers results in adaptive or heritable immunity. Therefore, upon reinfection or exposure to complementary sequences, the CRISPR array is transcribed and processed into short non-coding CRISPR RNAs (crRNA), which form a complex with the Cas proteins and targets the invading complementary sequences to mediate their cleavage (Fineran and Charpentier, 2012).

Given the essential roles of the CRISPR-Cas system in bacterial defense against phage infection and the deleterious effect of horizontal gene transfer (HGT), its expression and maintenance are controlled by factors in the cell in response to biotic and abiotic factors in the bacterial external environment (Westra et al., 2015; Alseth et al., 2019). So far, a few regulators such as the cAMP receptor protein (CRP), H-NS (Agari et al., 2010; Patterson et al., 2015), and LeuO (Westra et al., 2010), and environmental factors such as membrane stress (Perez-Rodriguez et al., 2011), temperature (Høyland-Kroghsbo et al., 2018), and metabolic stress (Yang et al., 2014; Patterson et al., 2015), and nutrient availability (Westra et al., 2015) has been associated with the regulation of the CRISPR-Cas system. Noticeably, the Crp can modulate the CRISPR-Cas system either as a positive or negative regulator depending on the host bacterial species. In *Escherichia* coli, the cAMP–Crp complex, which controls catabolite repression (Saier Jr and Ramseier, 1996), acts to inhibit the transcription expression of the CRISPR-Cas system (Yang et al., 2014), whereas in *Thermus thermophiles* and *Pectobacterium atrosepticum* the cAMP–Crp complex functions as a positive regulator for the regulation of the CRISPR-Cas system (Agari et al., 2010; Patterson et al., 2015).

Several Pseudomonas species possess the type I-F CRISPR-Cas system made up of 6 *cas* genes flanked by two CRISPR arrays (Wiedenheft et al., 2011). The CRISPR-Cas system in Pseudomonas aeruginosa PA14 is inducible, with its expression and function dependent on biotic and abiotic factors such as temperature, microbial interactions, phage exposure, nutrient availability, and population density (Høyland-Kroghsbo et al., 2017, 2018). The Type I-F CRISPR-Cas system in *P. aeruginosa* is regulated by the AHL QS system, where the *Cas* genes are significantly upregulated at high cell density but repressed by about 50% when the *lasI* and *rhlI* genes encoding biosynthesis of AHL signals were deleted (Høyland-Kroghsbo et al., 2017). The population density-dependent regulation of the CRISPR-Cas system is particularly vital as it allows the bacteria to defend against phage infection and the detrimental effects of horizontal gene transfer at a high cell density where it is vulnerable to phage infection (Abedon, 2012; Høyland-Kroghsbo et al., 2017).

In *P. aeruginosa*, the population density-dependent regulation of the CRISPR-Cas system is mediated particularly by the acyl-homoserine lactone (AHL) quorum sensing (QS) systems, the *las*, and *rhl* (Høyland-Kroghsbo et al., 2017). In this bacterium, the QS system is hierarchically organized, with the *las* on top of the hierarchy controlling the expression of the *pqs*, which in turn positively regulates the *rhl* systems (Lee and Zhang, 2015). The *las* system also directly regulates some *rhl*-controlled genes as both systems share overlapping regulon (Dekimpe and Déziel, 2009; Luo et al., 2015; Kostylev et al., 2019). In the *las* system, the transcriptional regulator LasR controls the expression of the autoinducer synthase LasI, which produces 3OC12HSL (*N*-3-oxo-dodecanoyl-L-homoserine lactone, OdDHL). The *rhl* system includes the autoinducer synthases RhlI, which produces the C4HSL (*N*-butanoyl-L-homoserine lactone). Expression of the *rhlI* is the under control of the transcriptional regulator RhlR. The *pqs* system produces the quinolone signal PQS (2-heptyl-3-hydroxy-4 (1*H*)-quinolone) encoded by the *pqs* gene cluster and regulated by MvfR (Ahator and Zhang, 2019). The regulation of the QS system in *P. aeruginosa* is influenced by environmental cues and cross-talk from other global regulators in the bacteria. One global regulator involves in cross-talk with the QS system is the Virulence factor regulator (Vfr) which positively regulates the LasR and RhlR. The Vfr-mediated induction of the AHL QS regulators is an essential part of the QS regulatory cascade as the autoinducer synthases LasI and RhlI depend on their cognate regulators for maximal expression (Albus et al., 1997).

The *P. aeruginosa* Vfr is a homolog of the Escherichia coli cAMP regulatory protein (CRP). However, Vfr does not function in catabolite repression control as seen in *E. coli* or other bacteria (Suh et al., 2002). Under the Vfr regulon, the expression of virulence genes is either indirectly *via* the QS system or through direct interaction with the Vfr binding sites in the promoter region of the genes. Such virulence factors include pyocyanin, elastase, exotoxin A, protease, type IV pili, and the type III secretion system (T3SS) (West et al., 1994; Albus et al., 1997; Fuchs et al., 2010; Berry et al., 2018). The function of Vfr is reported to depend on environmental factors such as calcium availability which influences the production of cAMP in *P. aeruginosa* (Beatson et al., 2002; Wolfgang et al., 2003b). The second messenger cAMP is an allosteric activator of Vfr. However, in *P. aeruginosa* both cAMP-dependent and -independent Vfr regulation of the *las* QS system has been detected (Fuchs et al., 2010), which shows that Vfr may be involved in other roles in *P. aeruginosa via* a cAMP-independent pathway.

*P. aeruginosa* thrives in diverse environments with varying levels of nutrients and trace elements, where they are outnumbered by phages. Given the importance of the CRISPR-Cas system for defense against phage infection and HGT, it was speculated that other endogenous and environmental factors were involved in regulating the CRISPR-Cas system. In this study, a genome-wide transposon screen was performed to identify regulators of the CRISPR-Cas system in *P. aeruginosa*. By exploiting the inducible property of the CRISPR-Cas system and the random insertion property of transposons, inactivation of the virulence factor regulator (Vfr) was identified to reduce cas expression. Further analysis showed that Vfr could modulate the expression of the type I-F CRISPR-Cas system *via* QS-dependent and -independent pathways. This regulatory cascade is vital for the CRISPR-Cas interference of HGT and the acquisition of spacers.

## Experimental procedures

### Culture conditions, strains, and plasmids

Supplementary Table S1, S2 list all the strains, plasmids and oligonucleotides used in this study. *P. aeruginosa* strain PA14 and mutants were grown at 37°C in tryptic soy broth (TSB) supplemented with 5 mM CaCl_2_ (Sigma) for calcium-rich media and or 5 mM EGTA (Sigma) for calcium-depleted media as indicated. Exogenous QS (AHL) molecules were added at a final concentration of 10 μM OdDHL (Sigma) + 50 μM BHL (Sigma) when necessary. For *P. aeruginosa* strains, carbenicillin, tetracycline, and gentamicin were added to the media at a final concentration of 300, 50, and 30 μg/ml, respectively, when needed. For *E. coli* strains, carbenicillin, tetracycline, and gentamicin were added to the media when necessary, at a final concentration of 200, 10, and 5 μg/ml, respectively. The plasmid pUCPT, a derivative of pUCP19 containing the *oriT* fragment from pK18mobsacB, which enhances transfer from *E. coli* Si17 to *P. aeruginosa* was used as the CRISPR non-targeted plasmid control, and for the construction of CRISPR-Cas targeted plasmid following the methods described previously with minor modifications (Patterson et al., 2015). The CRISPR 2 spacer1 fragment from *P. aeruginosa* PA14 was inserted into the HindIII/EcoRI multiple cloning sites of pUCPT to generate the CRISPR-targeted plasmid, pUCTSp1. The CRISPR-Cas targeted constructs pUCPTSp2n and pUCPTSp4n were created by inserting the CRISPR 2 spacer1 fragment with 2 and 4 nucleotide substitutions, respectively, into the HindIII/EcoRI multiple cloning sites of pUCPT using the ClonExpress MultiS One Step Cloning Kit (Vazyme Biotech). Sequences were verified by PCR and DNA sequencing using the M13F and M13R primers.

### In-frame deletion and integrative P*cas1-lacZ* reporter construction

To create chromosomal in-frame deletion in *P. aeruginosa* strains, the upstream and downstream DNA fragments flanking the gene of interest were amplified and ligated with the EcoRI/HindIII-digested pK18mobsacB using the ClonExpress MultiS One Step Cloning Kit (Vazyme Biotech). For chromosomal integrative *cas1*-*lacZ*, the up and down DNA fragments flanking the ATG of *cas1* and the *lacZ* gene were amplified with primers stated in Supplementary Table S2 and ligated with the EcoRI/HindIII-digested pK18mobsacB using the ClonExpress MultiS One Step Cloning Kit (Vazyme Biotech). The ligation products were transformed into *E. coli* DH5α and positive colonies selected by colony PCR and DNA sequencing. The correct constructs with the right fragment orientation were transformed into *E. coli* S17-1λ for conjugation with *P. aeruginosa* strains. Transconjugants were selected on minimal medium (MM) [0.2% (w/v) (NH_4_)_2_SO_4_ (Sigma), 0.41 mM MgSO_4_ (Sigma), 0.2% (w/v) mannitol (Oxoid), 40 mM K_2_HPO_4_ (Sigma), 14.7 mM KH_2_PO_4_,(Sigma) 32.9 μM FeSO_4_,(Sigma), 90 μM CaCl_2_,(Sigma), 16 μM MnCl_2_ (Sigma)(pH 7.2)] containing gentamicin (30 μg/ml), followed by selection of in-frame deletion mutants on MM supplemented with sucrose (Sigma) (10% w/v). Mutants were further confirmed by PCR and DNA sequencing.

### Transposon mutagenesis

The Mariner transposon, pBT20 was transferred from *E. coli* S17 to PA14 carrying the construct pMEPcas1-lacZ by conjugation. The resulting mating spot was scrapped and resuspended in 500 ml MM from which aliquot of serial dilution (10^−3^) was spread on MM agar (1.5% w/v) supplemented with Tetracycline, 50 μg/ml and X-gal [5-bromo-4-chloro-3-indoyl-D-galactopyranoside (Sigma)], 50 μg/ml. Single colonies of the transconjugants were picked onto the selection media composed of MM agar (1.5% w/v) and X-gal (50 μg/ml). Plates were incubated for 48 h and transposon mutants visually inspected for altered expression of the *Pcas1-lacZ* construct evident by the blue coloration of the colonies in comparison to wild-type. Colonies or transposon mutants with altered coloration were selected by colony tail PCR and DNA sequencing using primers listed in Supplementary Table S2 to map the position of transposon insertion through blast search against the *P. aeruginosa* UCBPP-PA14 genome.

### Qrt PCR

Cells were harvested after growth in specified media to OD_600_ = 1.5. RNA was extracted using the RNA extraction kit according to the manufacturer’s protocol (Qiagen). The quantity and integrity of the RNA was determined by Nanodrop and gel electrophoresis. One step Qrt PCR reaction were performed using the Tiangen One-step SYBR Green kit with the Applied Biosystems QuantStudio 6 Flex RT-PCR System.

### Electromobility shift assay

DNA promoter fragments for the EMSA probes were constructed by PCR using the indicated primers in Supplementary Table S2 and end-labeled with biotin using the biotin 3^I^ end DNA labeling kit (Thermo Fisher Scientific) as described in the kit protocol. EMSAs were performed using the LightShift chemiluminescent EMSA kit (Thermo Fisher Scientific) according to the kit protocol. Briefly, 1 nm of DNA fragments was incubated with cAMP-Vfr or Vfr and the binding buffer containing 1 μg/μL Poly (dI.dC), 50% Glycerol, 1% NP-40 1 M KCl 100 mM MgCl_2_ and 200 mM EDTA supplied with the kit for 25 min at 25°C. The cAMP was added at a concentration of 20 μM (Ferrell et al., 2008; Fuchs et al., 2010). The binding products were resolved on a 6% native polyacrylamide gel in 0.5X TBE and transferred to the nylon membrane at 380 mA (~100 V) for 30 min. The membrane was crosslinked at 120 mJ/cm^2^ for 45–60 s followed by detection of the biotin-labeled DNA by chemiluminescence using the Tanon 5,200 imaging system.

### Conjugation efficiency assay

The conjugation efficiency was performs using the method described by Patterson A.G. and colleagues (Patterson et al., 2015) with minor modification. The *E. coli* S17λ was used to transfer the CRISPR-targeted plasmids; pUCPTSp1, pUCPTSp2n, and pUCPTSp4n, and non-targeted plasmid, pUCPT into the *P. aeruginosa* strains through conjugation. Overnight cultures of *E. coli* and *P. aeruginosa* were mixed at a ratio of 1:1, washed twice and pellets resuspended in LB from which 50 μl were spotted on LB agar gently to prevent splatter. The mating spot was allowed to dry and incubated for 16 h at 37°C. The mating spot was scrapped completely and resuspended in 250 μl TSB from which serial dilutions of 10^−5^ were platted on TSB + EGTA and TSB + CaCl_2_ agar supplemented with carbenicillin (200 μg/ml). The conjugation efficiency was calculated as the ratio of transformants with the targeted plasmid compared with the transformants with the non-targeted plasmid.

### Plasmid loss and spacer acquisition

The plasmid loss and spacer acquisition assay was performed using a method described by Patterson A.G. and colleagues (Patterson et al., 2015) with modifications. The non-targeted plasmid pUCPT and the CRISPR2 spacer1-targeted plasmid, pUCPTSp1 were used to test CRISPR-Cas mediated interference assay. The plasmids were transferred to *P. aeruginosa* by mating with *E. coli* S17. Selected colonies were cultured overnight in 5 ml TSB with or without 5 mM EGTA or 5 mM CaCl_2_ and passaged for 5 days by sub-culturing 20 μl into 5 ml of fresh media. Each passage was serially diluted and 10^−6^ dilutions plated on LB with or without carbenicillin to count colonies that retain the plasmid. The CRISPR-Cas targeted constructs pUCPTSp2n and pUCPTSp4n, which contain a protospacer similar to CRISPR2 spacer1 with adaptation-priming mutations of 2 and 4 nucleotide substitutions, respectively, were used for the adaptation assay. *P. aeruginosa* strains initially transformed and passaged with pUCPTSp2n were further transformed with pUCPTSp4n to assay for primed adaptation. The genomic DNA from the samples of the final passage was extracted for the identification of expanded CRISPR2 arrays using primers stated in Supplementary Table S2. PCR products were resolved by 3% agarose gel electrophoresis to detect expansion of the CRISPR array.

### Beta-galactosidase assay

*Pseudomonas aeruginosa* cells were grown under specified culture conditions to appropriate time point and optical density (OD_600_). Briefly, 200 μl of cells was removed, pelleted and supernatants removed completely. Subsequently, 200 μl of Z buffer (8.52 g Na_2_HPO_4_, 5.5 g NaH_2_PO_4_.H_2_O, 0.75 g KCl and 0.246 g MgSO_4_.7H_2_O, pH 7.0), 20 μl 0.1% SDS and 20 μl chloroform was added and vortexed for 3 min. A volume of 200 μl ONPG (ortho-Nitrophenyl-β-galactoside) (Sigma), 4 mg/ml (dissolved in Z buffer) was added to the reaction mix and incubated at 37°C for a specified period of time. Finally, 600 μl of 1 M Na_2_CO_3_ was added to stop the reaction and the absorbance measured at A420nm. The blank sample was composed of 200 μl Z buffer; 200 μl ONPG and 600 μl Na_2_CO_3._ β-galactosidase activity was calculated as (1,000 × A_420_)/ (OD_600_ × Volume (ml) × time of reaction (min)) and expressed as miller units (MU) as previously described (Miller, 1972).

### Vfr protein expression and purification

The vector pET-28b (+) was used for Vfr protein expression. The Vfr coding sequence was amplified using the primer pairs listed in Supplementary Table S2 and ligated with the pET-28b (+) resulting in C-terminal His-tagged fusion. The resulting construct pET-VfrHis was transformed into *E. coli* BL21 (DE3) and grown in LB broth supplemented with kanamycin at 37°C to OD_600_ = 0.5 followed by induction with isopropyl β-D-thiogalactoside (IPTG) (Invitrogen) (0.5 mM) at 16°C overnight. Bacterial pellets obtained were resuspended in ice-cold lysis buffer [50 mM NaH_2_PO4 (Sigma), 300 mM NaCl (Sigma), 1 mM DTT (Sigma), 10 mM imidazole (Sigma), pH 7.5] containing protease inhibitors (Complete mini, EDTA free, Roche) and lysed by sonification. Cell-free supernatants incubated with ProteinIso Ni-NTA Resin (TransGene Biotech, China) at 4°C for 2 h. Subsequently, the resins were washed 4 times with wash buffer (50 mM NaH_2_PO4, 300 mM NaCl, 1 mM DTT, 50 mM imidazole, pH 7.5) and the proteins eluted with the elution buffer (50 mM NaH_2_PO4, 300 mM NaCl, 1 mM DTT, 300 mM imidazole, pH 7.5). The protein purity was determined by SDS-PAGE analysis (Supplementary Figure S1C) and dialyzed against the PBS buffer (PBS, 5% glycerol, pH 7.4) at 4°C.

### Intracellular cAMP assay

Quantification of intracellular cAMP was performed by adapting the method used by Fulcher et al. (2010). Bacteria were grown in TSB supplemented with EGTA or CaCl_2_ to OD_600_ = 1.0. Briefly, 1 ml of bacterial culture was centrifuged at 12,000 *rpm* for 2 min at 4°C and the pellets washed twice with 1 ml of ice-cold 0.9 M NaCl. The cells were lysed by resuspension in 100 μl of 0.1 N HCl, incubated on ice for 15 min with intermittent agitation every 5 min. The lysates were centrifuged at 12,000 rpm for 5 min at 4°C and the cell-free supernatant obtained for cAMP quantification using the cAMP enzyme immunoassay kit (Sigma-Aldrich) as per the manufacturer’s recommendation for sample acetylation. For protein determination, duplicate samples were suspended in 100 μl of ice-cold phosphate buffered saline (PBS), lysed by 3 freeze–thaw cycles and centrifuged at 1,200 rpm for 5 min. The protein concentration was determined by the Pierce BCA protein assay kit (Thermo Fisher Scientific). The intracellular cAMP values were presented as pmole per μg of total protein.

## Results

### Mutations in Vfr reduce *Cas* gene expression

**Figure 1:**
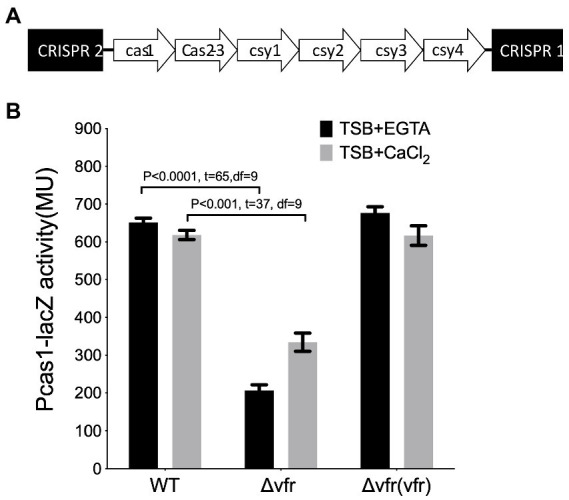
Identification of Vfr as a regulator of Type 1F CRISPR-Cas system **(A)**. Organization of the type I-F CRISPR-Cas system in *Pseudomonas aeruginosa* UCBPP-PA14 **(B)**. The β galactosidase assay showing the expression of *cas1* in the wild-type PA14, and *vfr* in-frame deletion mutants, *and the vfr complement*, *Δvfr (vfr)*, grown in calcium-deplete (TSB + CaCl_2_) and calcium-rich (TSB + CaCl_2_) media. Data represent mean ± SD from three independent repeats. Statistical analysis was conducted by student’s *t*-test with a *p* value of <0.05 is considered significant.

In this study, the *Pseudomonas aeruginosa* UCBPP_PA14, which contains the type I-F CRISPR-Cas system with six *cas* genes flanked by two CRISPR arrays (Figure 1A), was the wild-type (WT) strain used. Screening for the potential regulators of the CRISPR-Cas system was performed by genome-wide random transposon mutagenesis with the mariner transposon in the WT expressing the P*cas1*-*lacZ* reporter construct. The lacZ gene was placed under the control of the *cas1* promoter to identify transposon insertion sites that result in the downregulation of *cas1* promoter activity. Colonies with downregulated P*cas1*-*lacZ* expression resulting in lighter blue color to the WT parental mating strains were selected for transposon insertion mapping (Supplementary Figure S1A). Of particular interest among the transposon mutants identified from the screening was the insertion into the genes encoding the Virulence factor regulator (Vfr) (Supplementary Figure S1A). Additional bioassay of the *cas1* promoter in the *vfr* transposon mutant showed a reduction in the activity of the promoter (Supplementary Figure S1B).

Since the secondary messenger cAMP required for Vfr function is dependent on calcium availability (Dasgupta et al., 2006), the effect of Vfr on the Cas gene expression was examined in calcium-rich and calcium-depleted media. Using the in-frame deletion mutant showed that Vfr positively regulates the *Cas* gene expression under both calcium depleted and calcium-rich conditions (WT/∆vfr in TSB + EGTA: *p* < 0.0001, *t* = 65, df = 9; WT/∆vfr in TSB + CaCl_2_: *p* < 0.0001, *t* = 37, df = 9) (Figure 1B; Supplementary Figure S2C). This shows that Vfr can regulate the CRISPR-Cas system in the presence or absence of cAMP, which is not surprising as the cAMP-independent functionality of Vfr is possible in *P. aeruginosa* (Fuchs et al., 2010).

### Vfr binding site is required for direct regulation of the CRISPR-Cas system

**Figure 2:**
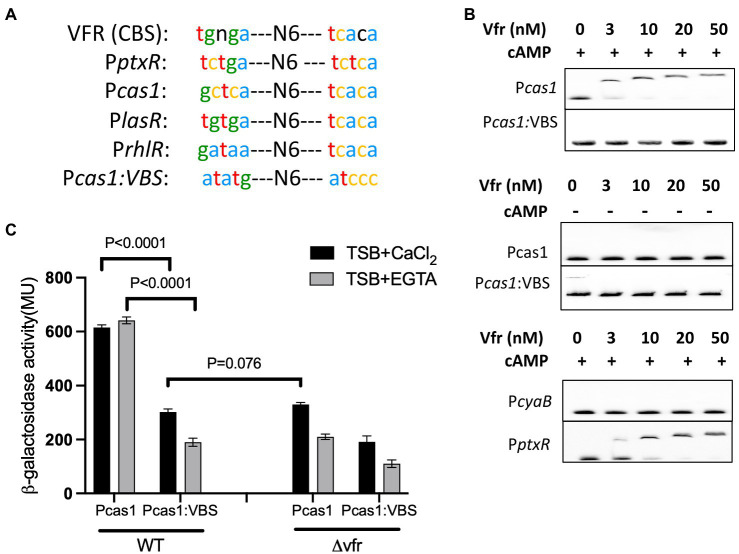
Vfr directly regulates the CRISPR-Cas system *via* a cAMP-dependent pathway. **(A)** The Vfr-binding consensus binding site [Vfr (CBS)] in *P. aeruginosa* and the Vfr binding sites in the promoter region of selected Vfr-regulated gene, *ptxR*, *lasR*, *and rhlR* share similarities with that of the *cas1* promoter (P*cas1*). The bottom sequence is an altered P*cas1* Vfr binding sequence (Pcas1:VBS), which was generated to investigate Vfr binding and regulation of *cas1*. **(B)** EMSA analysis of Vfr with the *cas1* promoter and its derivative Pcas1 (VBS) with an altered Vfr binding site. The probes of *cyaB* and *ptxR* promoter were used as negative and positive controls, respectively. Different Vfr concentrations were used as indicated with or without 10 μM cAMP. **(C)** Expression of the P*cas1* promoter construct composed of intact and altered VBS-binding sites in WT and *vfr* mutant grown in TSB + CaCl_2_ and TSB + EGTA. Data shown are the mean ± SD (*n* = 6). Statistical analysis was conducted by student’s *t*-test with a *p* value of < 0.05 is considered significant.

The Virulence factor regulator, Vfr in *P. aeruginosa* recognizes the nucleotide sequence “tgnga-N6tcaca” in its target promoter region as the binding site. Typically, Vfr binding sites show a high degree of variability at the left palindromic sequence portion (Fuchs et al., 2010; Figure 2A). Nucleotide sequence alignment revealed the sequence “gctca N6 tcaca” in the promoter region of *cas1*, which shares similarity with the conserved binding sequence of Vfr (Figure 2A) in known Vfr-regulated genes in *P. aeruginosa* (Figure 2A; Supplementary Figure S3; Yahr and Wolfgang, 2006; Ferrell et al., 2008; Fuchs et al., 2010). This implies that Vfr could regulate *cas1* expression through protein-promoter interaction. Since Vfr regulates its target genes *via* interacting with specific sequences (*Vfr* box) in the presence of the second messenger cAMP (West et al., 1994), an EMSA analysis was used to investigate the interaction between Vfr and the nucleotide sequence of the *cas1* promoter (P*cas1*). The EMSA analysis showed an interaction between the Vfr and the P*cas1* DNA fragment occurred when cAMP was added to the reaction mix (Figure 2B). By mutating the putative Vfr binding sequence in Pcas1 to “atatg N6 atccc,” Vfr did not interact with the Pcas1 fragment with and without cAMP (Figure 2B). Furthermore, the Vfr interaction was examined using the DNA probe from the promoter region of the *cyaB* (P*cyaB*), a major intracellular cAMP synthase in *P. aeruginosa* which lacks the Vfr binding site as a negative control (Smith et al., 2004; Topal et al., 2012) and the DNA probe from the promoter region of *ptxR* (P*ptxR*), a Vfr-regulated transcriptional regulator as a positive control (Ferrell et al., 2008). Vfr showed interaction with the P*ptxR* but not P*cyaB* (Figure 2B).

To further investigate the *cas1* gene expression, the transcriptional *lacZ* fusion constructs composed of the promoter region of the *cas1* gene with intact (Pcas1) and mutated Vfr binding site (Pcas1:VBS) were constructed and transformed into the WT and *vfr* mutant (∆*vfr*). In the WT, the expression of the *cas1* promoter with the mutant Vfr binding site was reduced compared with the reporter containing the intact Vfr binding site (P*cas1*/ P*cas1*VBS expression in WT (+CaCl_2_): *p* < 0.0001, *t* = 31.47 df = 7.37; (–CaCl_2_): *p* < 0.0001, *t* = 43.64 df = 6.63)) (Figure 2C). In the ∆*vfr*, a similar effect of the P*cas1* expression from the intact Vfr binding site was observed as in the WT possessing the mutated Vfr binding site (WT (P*cas1*VBS)/∆*vfr* (P*cas1*) in (+CaCl_2_): *p* = 0.076, *t* = 2.95 df = 8.33; (–CaCl_2_): *p* = 0.069, *t* = 2.112 df = 7.79) (Figure 2C). Unexpectedly, in the *vfr* mutant, expression of *cas1* from P*cas1*:VBS construct significantly reduced compared to the expression from the P*cas1* construct under both calcium-rich and depleted conditions (∆*vfr* (P*cas1*/P*cas1*VBS) + CaCl_2_: *p* < 0.0001, *t* = 13.86 df = 8.221; –CaCl_2_: *p* < 0.0001, *t* = 9.70 df = 8.46)) (Figure 2C). These results demonstrate that Vfr and its binding site are required for activation of the *cas* operon and that Vfr can control activation of the *cas* genes *via* an alternative pathway.

**Figure 3:**
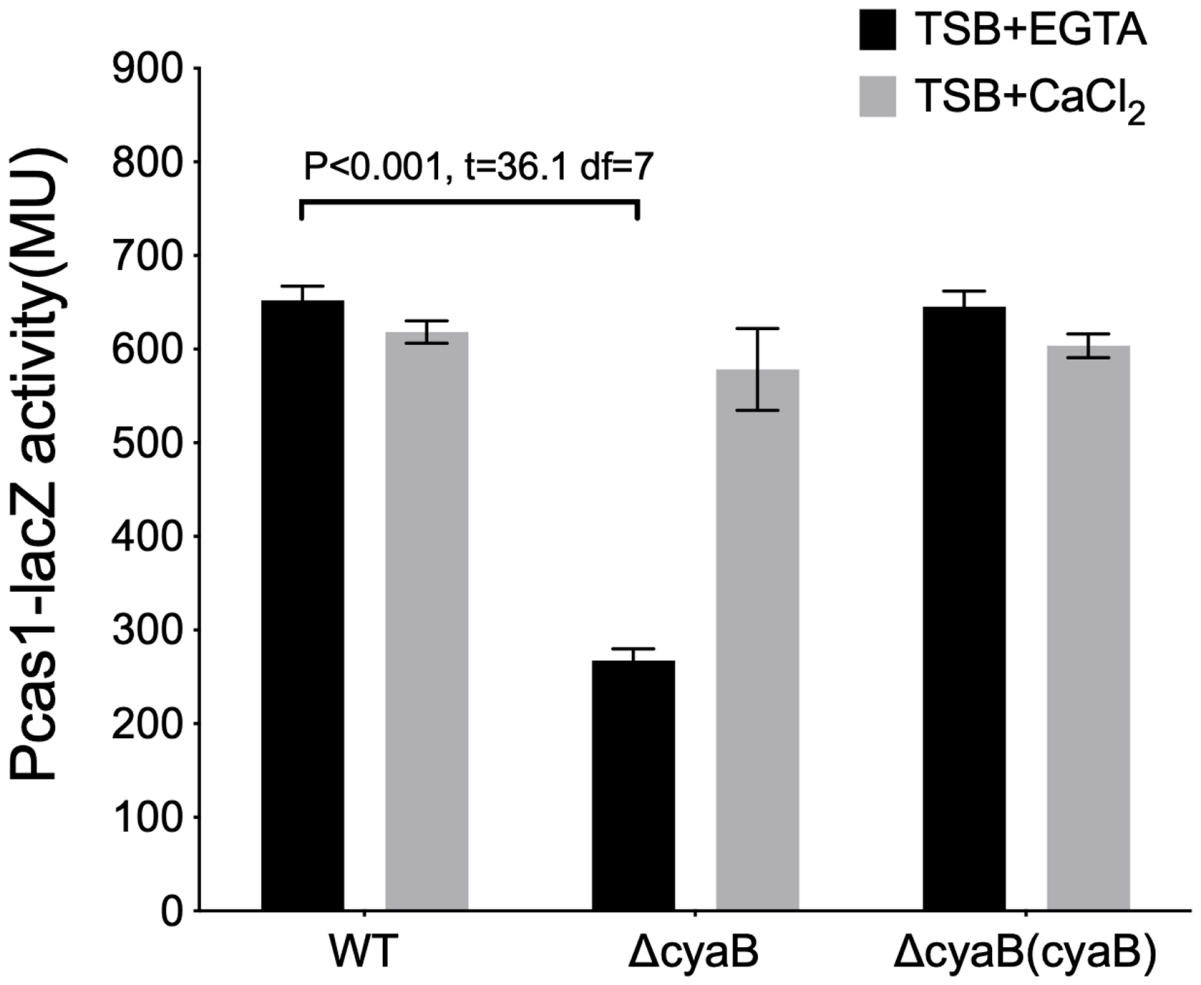
The cAMP production affects cas gene expression. The expression of *cas1* in the wild-type PA14, and *cyaB* in-frame deletion mutants, *ΔcyaB and the cyaB complement*, *ΔcyaB (cyaB)*, grown in calcium depleted (TSB+ EGTA) and calcium-rich (TSB + CaCl_2_) media. Data represents the Mean ± SD of six independent experiments. Statistical analysis was conducted by student’s *t*-test with a *p* value of < 0.05 is considered significant.

Also, the deletion of *cyaB*, the major cAMP synthase in *P. aeruginosa* under calcium depleted conditions (Wolfgang et al., 2003b; Supplementary Figure S4), resulted in a reduction in the *cas* gene expression in calcium depleted media but not in the calcium-rich media (∆*cyaB*/WT in (+EGTA): *p* < 0.0001, *t* = 36.1 df = 7; in (+CaCl_2_): *p* = 0.055, *t* = 2.39 df = 5.8) (Figure 3). This implies that the second messenger cAMP influences *cas* gene expression under calcium-depleted conditions and that the Vfr regulation of the *cas* gene expression under calcium-rich conditions may occur *via* an alternative pathway independent of cAMP.

### Vfr regulates the CRISPR-Cas system *via* the AHL QS system

**Figure 4:**
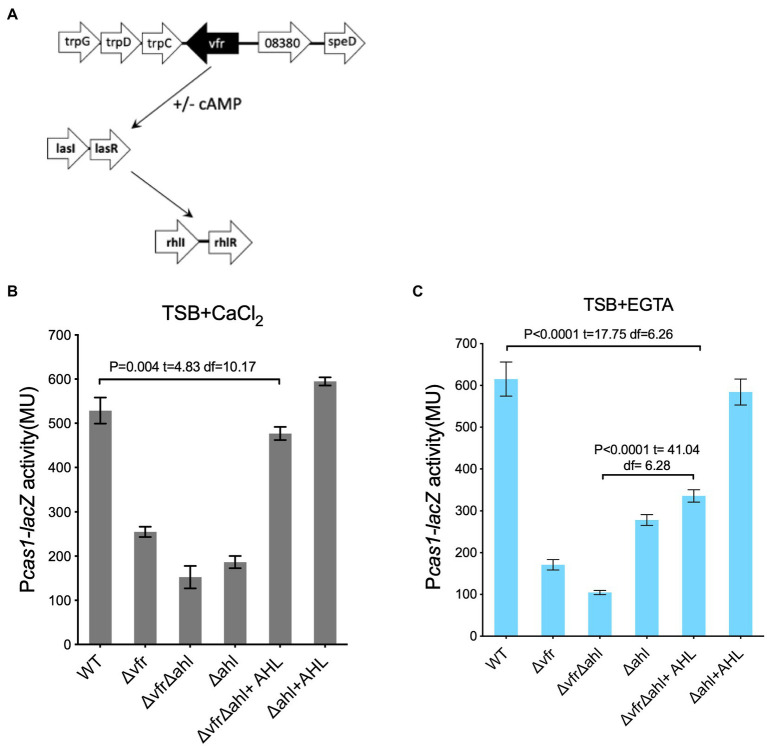
Vfr regulates the CRISPR-Cas system *via* AHL QS system. **(A)** Diagrammatic representation of Vfr and AHL QS regulation in *P. aeruginosa*. The expression of *cas1* in the WT and mutants *Δvfr*, *ΔlasIΔrhlI designated as (Δahl) and ΔvfrΔahl strains* grown in **(B)**, calcium-rich and **(C)**, calcium-rich media. Exogenous QS molecules (AHL) were added to the media as indicated. Data shown are the mean ± SD (*n* = 6). Statistical analysis was conducted by student’s *t*-test with a *p* value of < 0.05 is considered significant.

In *P. aeruginosa*, Vfr regulates the *las* QS system both in the presence and absence of cAMP (Figure 4A; Supplementary Figure S5; Albus et al., 1997; Fuchs et al., 2010). Prompted by the regulation of the CRISPR-Cas system by the hierarchically organized AHL QS system (Lee and Zhang, 2015; Høyland-Kroghsbo et al., 2017) and the presence of the *las*/*rhl* box in the promoter region of the *cas1* (Supplementary Figure S3), we hypothesized that the Vfr could indirectly regulate the CRISPR-Cas system *via* the AHL system in the absence of cAMP. To investigate this Vfr-QS-CRISPR regulatory cascade, the *cas* gene expression was examined in the double deletion *lasI* and *rhlI* mutant designated as “∆*ahl*” in the ∆*vfr* strain or WT background grown under calcium depleted (+EGTA) and calcium-rich (+CaCl_2_) conditions.

In both calcium depleted and calcium replete media, *cas1* expression was reduced in the ∆*vfr*∆*ahl*, ∆*ahl*, and ∆*vfr* strains compared to the WT (Figures 4B,C). Addition of exogenous AHL (10 μM OdDHL +50 μM BHL) to the ∆*ahl* strain rescued the expression of the *cas1* gene in both conditions (∆*ahl*/∆*ahl* + AHL in (+EGTA): *p* < 0.0001, *t* = 24.45 df = 6.67; ∆*ahl* + AHL /WT in (+EGTA): *p* = 0.14, *t* = 1.62 df = 9.45), however, *cas1* expression in the ∆*vfr*∆*ahl* strain was not fully rescued to the WT level when the media was supplemented with exogenous AHL (∆*vfr*∆*ahl* + AHL/WT in (+CaCl_2_): *p* = 0.004, *t* = 4.83 df = 10.17; (+EGTA): *p* < 0.0001, *t* = 17.75 df = 6.26) (Figure 4B). Due to the global regulon of the Vfr and the QS system, there is a possibility that other factors that are vital for complete regulation of the Vfr-QS-CRISPR regulatory cascade may be affected by the deletion of both Vfr and the AHL QS synthases. Taken together, these results show that the Vfr can regulate the CRISPR-Cas system in *P. aeruginosa* either directly or *via* the AHL QS system.

### Vfr influences plasmid retention and HGT interference

The CRISPR-Cas system facilitates the targeted degradation of invading genetic materials that share similarities with spacers located in the CRISPR array. The spacers in the CRISPR arrays are derived from the invaders and are essential for immunologic memory and defense against previously encountered foreign elements (Bhaya et al., 2011; Dy et al., 2014). Following the transcriptional control of the Vfr and the AHL QS system on the cas gene expression, the impact of the Vfr and QS on CRISPR-Cas mediated interference, spacer acquisition, and conjugation efficiency were further investigated.

**Figure 5:**
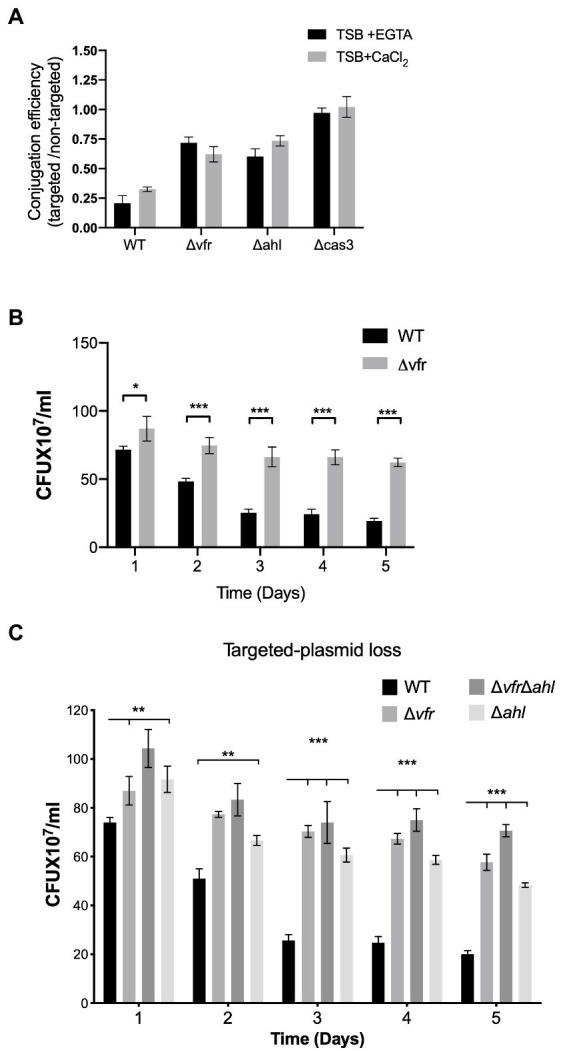
Vfr influences Crispr-Cas adaptation and HGT interference. **(A)** Conjugation efficiency of the WT and *Δvfr*, *Δahl and Δcas3* strains. The conjugation efficiency is calculated as the ratio of the CRISPR-Cas targeted and non-targeted plasmids in the individual strains following conjugation with *E. coli* S17 donor. The *P. aeruginosa* strains were grown in TSB + EGTA and TSB + CaCl_2_. Data shown are the mean ± SD of *n* = 6 experiments. Retention of CRISPR-Cas targeted plasmid in *P. aeruginosa* strains spread on LB containing carbenicillin for selection over 5-day passage in TSB + EGTA **(B)**, and TSB+ CaCl_2_ **(C)**. Plasmid loss was scored by counting positive colonies on plates. Data represent the mean ± SD (*n* = 6). Statistical significance was calculated using Bonferroni-Dunn method multiple comparison test (*p ≤ 0.01, **p ≤ 0.001, and ***p ≤ 0.0001).

Using the *E. coli* S17 as a donor, the WT, ∆*vfr*, ∆*ahl*, and ∆*cas3* strains were transformed with the CRISPR-targeted plasmid containing a spacer with the GG PAM recognized by CRISPR2 spacer 1 (pUCPTSp1) and the non-targeted plasmid (pUCPT) devoid of spacers recognized by the CRISPR-Cas system. The conjugation efficiency was calculated as the ratio of the colonies retaining the CRISPR-targeted plasmid to that of the non-targeted plasmid. In the ∆*cas3* strain, which is defective in CRISPR-mediated interference and spacer acquisition (Høyland-Kroghsbo et al., 2017), the conjugation efficiency of the targeted plasmid was comparable to the non-targeted plasmid under both conditions tested (Figure 5A). The WT showed the least conjugation efficiency in comparison to the ∆*vfr* ((+EGTA): *p* < 0.0001, *t* = 10.75 df = 5; (+CaCl_2_): *p* < 0.0001, *t* = 15.52 df = 9) and ∆*ahl* (+CaCl_2_): *p* < 0.0001, *t* = 10.62 df = 9; (+EGTA): *p* < 0.0001, *t* = 21.31 df = 7) strains under both calcium-rich and calcium depleted conditions (Figure 5A), showing that in the absence of the Vfr and the AHL QS system, the CRISPR-mediated interference of plasmid transfer is reduced.

Next, the impact of Vfr and AHL QS on CRISPR-mediated plasmid loss was tested by transforming the CRISPR-targeted and non-targeted plasmids into the *P. aeruginosa* strains followed by a 5-day successive passage in calcium-depleted and calcium-rich media. Aliquots of serial dilutions from each passage were plated on LB agar supplemented with carbenicillin and X-gal. The plasmid loss was assessed by counting the colonies that grew on the plates following overnight incubation at 37°C. Under both calcium-rich and calcium-depleted conditions, over 5 days of passage, the ∆*vfr*, ∆*ahl*, and ∆*vfr*∆*ahl* showed significantly increased retention of the CRISPR-targeted plasmid compared to the WT (Figures 5B, C). However, the retention of the non-targeted plasmid was similar in the WT, ∆*vfr*, ∆*ahl*, and Δ*vfr*Δ*ahl* strains (Supplementary Figure S6).

**Figure 6:**
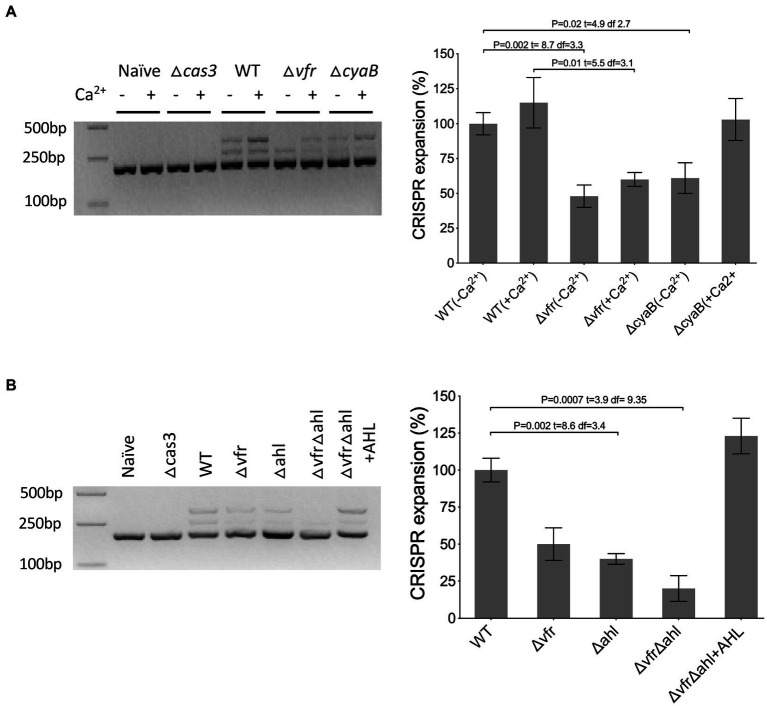
Vfr regulates CRISPR-Cas spacer acquisition. **(A**,**B)** Acquisition of spacers in the CRISPR2 locus was identified by PCR of transconjugants after 5 days of passage with the CRISPR-targeted plasmid. As a negative control, wild-type and *cas3* mutant were transformed with the non-targeted plasmid (naïve) and targeted plasmid, respectively. The adaptation events are signified by expansion of the arrays and 60 bp extension of the CRISPR locus. The media for *ΔvfrΔahl* passage was supplemented with Exogenous QS molecules (+AHL). Figure presented is representative of three identical repeats. The intensity of the bands was quantified by image J software. The WT mean is normalized to 100%. Statistical analysis was conducted by student’s *t*-test with a *p* value of < 0.05 is considered significant.

The CRISPR-Cas system builds immunological memory against previously encountered genetic elements by incorporating the target sequence into the CRISPR array, which results in the expansion of the array. To investigate the impact of Vfr and the AHL QS system on the expansion of the arrays, a PCR reaction targeting the CRISPR2 array, which has a higher frequency of adaptation (Westra et al., 2015; Høyland-Kroghsbo et al., 2017) was performed. The plasmids pUCPTsp2n and pUCPTSp4n containing a protospacer similar to CRISPR2 spacer1 with adaptation-priming mutations were transformed into the *P. aeruginosa* strains and assayed for the expansion of the CRISPR2 locus in the colonies passaged in calcium-rich and calcium depleted media. In the absence of *cas3*, no expansion of the CRISPR array occurred under both conditions tested (Figure 6). Also, the WT containing the naïve plasmid with no protospacer targeted by the CRISPR-Cas system did not induce the expansion of the CRISPR array (Figure 6). In the WT there was an expansion of the arrays with the incorporation of spacers when passaged in both calcium-rich and depleted conditions. In comparison with the WT, expansion of the CRISPR array was less in the ∆*vfr* strain when passaged in the calcium-depleted medium compared to the calcium-rich medium (Supplementary Figure S7A). As expected, expansion of the CRISPR array was reduced in the ∆*cyaB* when passaged in calcium-depleted conditions compared to calcium-rich conditions (*p* < 0.022, *t* = 3.8 df = 3.7). Spacer acquisition in the ∆*vfr*∆*ahl and* ∆*ahl* strains was significantly reduced, however, the addition of exogenous AHLs to the ∆*vfr*∆*ahl* strain rescued spacer acquisition to the WT level (Supplementary Figure S7B). In general, these results demonstrate that the Vfr and the AHL QS system are required for CRISPR-mediated spacer acquisition and interference of HGT in *P. aeruginosa*.

## Discussion

The CRISPR-Cas immune system is a defense mechanism for most bacteria and archaea against phage infection and acquiring deleterious genetic elements *via* HGT. Despite the remarkable advancements in genetic engineering achieved by repurposing the CRISPR-Cas system, the molecular mechanisms by which bacteria regulate this defense mechanism are largely unknown. Identifying the regulatory factors that control the CRISPR-Cas system in bacteria will provide a platform for understanding its role in bacterial lifestyle and exploitation for controlling bacterial infections. Here, using *Pseudomonas aeruginosa* and the type I-F CRISPR-Cas system, the global transcriptional regulator Vfr is shown as a regulator of the CRISPR-Cas immune system. The deletion of *vfr* reduces the *cas* gene expression and attenuates CRISPR-mediated defense mechanisms such as the spacer acquisition and interfering with previously encountered genetic elements. This work provides evidence that Vfr can directly or indirectly control the transcription of the genes required for the maintenance CRISPR-Cas system function depending on the availability of calcium in the local environment. Furthermore, the Vfr regulatory cascade is shown to act with or without the second messenger cAMP in controlling the CRISPR-Cas immune system *via* an alternative pathway that involves the AHL QS systems, *lasIR*, and *rhlIR*.

Given the previously known functions of Vfr involve interaction with the allosteric activator, cAMP, whose biosynthesis is induced by calcium limitation (Wolfgang et al., 2003b), this work highlights an alternative pathway for Vfr and the type I-F CRISPR-Cas system regulation in *P. aeruginosa* dependent on calcium availability. The Vfr-cAMP complex promotes direct regulation of the CRISPR-Cas system under calcium-depleted conditions whereas, under calcium-rich conditions, the AHL QS system mediates the Vfr-CRISPR-Cas regulatory cascade (Figure 4C). The ability of Vfr to regulate the QS system in calcium-rich conditions where cAMP levels are reduced is contrary to the previous reports that Vfr is functionally dependent on its allosteric regulator cAMP (Wolfgang et al., 2003b). Prompted by the AHL QS regulation of the CRISPR-Cas system in *P. aeruginosa* (Høyland-Kroghsbo et al., 2017), we investigated an alternative pathway under calcium-rich conditions, where Vfr regulates the expression of the CRISPR-Cas system *via* the AHL QS system (Figure 4, Supplementary Figure S5). In support of this regulatory cascade, Vfr controls the expression of the AHL QS genes under both calcium-rich and calcium–deplete conditions (Supplementary Figure S5). Also, double deletion of the *vfr* and AHL synthases (∆*vfr*∆*ahl*) significantly reduced *cas* gene expression (Figure 4), HGT interference (Figure 5), and spacer acquisition (Figure 6) compared to the ∆*vfr* and ∆*ahl* single mutants under calcium-rich conditions.

The identification of Vfr as a regulator of the CRISPR-Cas system further expands the functional spectrum of the global regulator in *P. aeruginosa*. Vfr is a Crp-family transcriptional regulator and shares a similar binding site as the cAMP receptor protein (Crp) of *E. coli*, however, it is not functionally complementary with Crp and is not involved in carbon catabolite regulation as observed in *E. coli* (West et al., 1994). In *P. aeruginosa*, Vfr regulates quorum sensing, pyocyanin, elastase, and exotoxin A production (West et al., 1994; Albus et al., 1997). Transcriptome analysis showed that deletion of *vfr* results in decreased expression of over 200 genes, including those encoding the type III secretion system, type IV pilus biogenesis, and type II secretion (Wolfgang et al., 2003b). This work, therefore, extends the function of Vfr from a regulator of virulence factors (offense) to a regulator of an adaptive immune system (defense), which ensures protection of the bacterial cells while competing for survival under conditions where it is prone to phage infection and harmful effects of HGT.

Acquiring genetic materials *via* HGT has added benefits for bacteria as it drives evolutionary adaptive traits such as antibiotic resistance, virulence, and adaptation to environmental stress conditions (Vogan and Higgs, 2011). This implies that constitutive expression of the CRISPR-Cas system will not be overall beneficial as the bacteria may lose out on the benefits of HGT. Controlled expression of the CRISPR-Cas system in the bacteria will thus allow the acquisition and incorporation of beneficial genetic elements. Also, a hyperactive CRISPR-Cas system runs the risk of autoimmunity, which can be particularly deleterious to the bacterial population (Stern et al., 2010; Høyland-Kroghsbo et al., 2017). Hence, the induction of the CRISPR-Cas system by multiple pathways observed in the Vfr-QS-CRISPR regulatory cascade in response to specific environmental factors will be more beneficial to the bacterial population.

The Vfr is hierarchically above the AHL QS system and shares regulon with the *las* and *rhl* systems which make up over 20% of *P. aeruginosa* genes (Coggan and Wolfgang, 2012; Ahator and Zhang, 2019). It is unknown if other factors under the Vfr and QS regulon may account for the inability of exogenous AHL to rescue the *Cas* gene expression in the ∆*vfr*∆*ahl* strains (Figures 4B,C) but restore its ability to incorporate spacers into its CRISPR array (Figure 6B). Identifying such factors will help understand the gamut of the Vfr-QS-CRISPR regulatory network.

In the *cas1* promoter region, the Vfr box partially overlaps with one of the *las*/*rhl* box identified using the Prodoric database (Supplementary Figure S3; Münch et al., 2003), which may account for the reduced expression of *cas1* from the construct with altered Vfr binding site (Figure 2). This partial overlap in binding sites reveals the possibility of the Vfr and the AHL regulators competing for the binding site but rules out simultaneous binding at the overlapping site. Simultaneous binding at the other distant *las/rhl* boxes may be possible but not yet experimentally verified. How the bacteria coordinate the Vfr-QS-CRISPR regulatory cascade may depend on the combination of bacterial metabolic requirements and response to environmental factors. Despite the overlapping regulon, the Vfr regulates pili formation (Coggan et al., 2022), which serves as phage binding sites and entry portals for nucleic acid (Craig et al., 2004; Harvey et al., 2018).

Identification of Vfr as a central regulator of the CRISPR-Cas immune system might have significant implications for understanding bacterial physiology. The two mechanisms with which Vfr controls the transcriptional expression and function of the CRISPR-Cas immune system would enable the pathogen to activate the immune system against phage infections and HGT regardless of the changes in the bacterial quorum level or the local calcium concentrations, which could vary drastically under either *in vivo* or *in vitro* environmental conditions. For example, the decontrolled calcium homeostasis in the Cystic Fibrosis lung results in elevated calcium in body fluids (Broder et al., 2016). Similarly, wounding accompanies a surge in calcium concentrations from early in the post-wound period through the inflammatory and proliferative phases and the remodeling phase (Lansdown, 2002). Furthermore, the Vfr regulation of the CRISPR-Cas immune system might facilitate the design and development of effective and reliable phage therapy. Firstly, the Vfr is hierarchically above the AHL QS system in regulating the CRISPR-Cas system. Secondly, the Vfr-cAMP complex regulates factors such as type IV pili biogenesis which is not under the control of QS (Beatson et al., 2002). Aside from the roles in pathogenesis and biofilm formation, the type IV pili of *P. aeruginosa* is vital for transformation, conjugation, phage adsorption, and infections (50–52; Craig et al., 2004). Also, pili-mediated twitching motility increases the chances of phage-bacteria interactions due to the cell–cell aggregated movement, which creates a spatial vulnerability for phage interaction with the cells (Abedon, 2012; Alexandre, 2015). Therefore, targeting Vfr could have dual effects in safeguarding phage therapy by turning down the expression of the CRISPR-Cas phage immune system and avoiding the formation of pili, which serve as receptors and entry ports for phage particles (Craig et al., 2004). The diagrammatic representation of the Vfr-QS-CRISPR regulatory cascade under calcium-depleted and calcium-rich conditions is in Supplementary Figure S7.

## Data availability statement

The original contributions presented in the study are included in the article/Supplementary material, further inquiries can be directed to the corresponding author.

## Author contributions

SA and L-HZ designed the experiments. SA, YL, and JW conducted the experiments. SA, YL, JW, and L-HZ performed the data analysis. SA and L-HZ wrote the manuscript. All authors contributed to the article and approved the submitted version.

## Funding

This work was supported by the Guangdong Forestry Science and Technology Innovation Project (2020KJCX009) and Guangdong Technological Innovation Strategy of Special Funds (grant no. 2018B020205003). YL is supported by the China Scholarship Council (CSC) (Grant No. 202008440425).

## Conflict of interest

The authors declare that the research was conducted in the absence of any commercial or financial relationships that could be construed as a potential conflict of interest.

## Publisher’s note

All claims expressed in this article are solely those of the authors and do not necessarily represent those of their affiliated organizations, or those of the publisher, the editors and the reviewers. Any product that may be evaluated in this article, or claim that may be made by its manufacturer, is not guaranteed or endorsed by the publisher.

## References

[ref1] Abedon S. T. (2012). Spatial vulnerability: bacterial arrangements, microcolonies, and biofilms as responses to low rather than high phage densities. Viruses 4, 663–687. doi: 10.3390/v4050663, PMID: 22754643 PMC3386622

[ref2] Agari Y. Sakamoto K. Tamakoshi M. Oshima T. Kuramitsu S. Shinkai A. (2010). Transcription profile of *Thermus thermophilus* CRISPR systems after phage infection. J. Mol. Biol. 395, 270–281. doi: 10.1016/j.jmb.2009.10.057, PMID: 19891975

[ref3] Ahator S. D. Zhang L. (2019). Small is mighty-chemical communication systems in *Pseudomonas aeruginosa*. Annu. Rev. Microbiol. 73, 559–578. doi: 10.1146/annurev-micro-020518-120044 31226024

[ref4] Albus A. M. Pesci E. C. Runyen-Janecky L. J. West S. E. Iglewski B. H. (1997). Vfr controls quorum sensing in *Pseudomonas aeruginosa*. J. Bacteriol. 179, 3928–3935. doi: 10.1128/jb.179.12.3928-3935.1997, PMID: 9190808 PMC179201

[ref5] Alexandre G. (2015). Chemotaxis control of transient cell aggregation. J. Bacteriol. 197, 3230–3237. doi: 10.1128/JB.00121-15, PMID: 26216846 PMC4573731

[ref6] Alseth E. O. Pursey E. Luján A. M. McLeod I. Rollie C. Westra E. R. (2019). Bacterial biodiversity drives the evolution of CRISPR-based phage resistance. Nature 574, 549–552. doi: 10.1038/s41586-019-1662-9, PMID: 31645729 PMC6837874

[ref7] Beatson S. A. Whitchurch C. B. Sargent J. L. Levesque R. C. Mattick J. S. (2002). Differential regulation of twitching motility and elastase production by Vfr in *Pseudomonas aeruginosa*. J. Bacteriol. 184, 3605–3613. doi: 10.1128/JB.184.13.3605-3613.2002, PMID: 12057955 PMC135129

[ref8] Berry A. Han K. Trouillon J. Robert-Genthon M. Ragno M. Lory S.. (2018). cAMP and Vfr control exolysin expression and cytotoxicity of *Pseudomonas aeruginosa* taxonomic outliers. J. Bacteriol. 200, 135–158. doi: 10.1128/JB.00135-18, PMID: 29632090 PMC5971474

[ref9] Bhaya D. Davison M. Barrangou R. (2011). CRISPR-Cas systems in bacteria and archaea: versatile small RNAs for adaptive defense and regulation. Annu. Rev. Genet. 45, 273–297. doi: 10.1146/annurev-genet-110410-132430, PMID: 22060043

[ref10] Broder U. N. Jaeger T. Jenal U. (2016). LadS is a calcium-responsive kinase that induces acute-to-chronic virulence switch in *Pseudomonas aeruginosa*. Nat. Microbiol. 2:16184. doi: 10.1038/nmicrobiol.2016.184, PMID: 27775685

[ref11] Coggan K. A. Higgs M. G. Brutinel E. D. Marden J. N. Intile P. J. Winther-Larsen H. C.. (2022). Global regulatory pathways converge to control expression of *Pseudomonas aeruginosa* type IV. MBio 13, e03696–e03621. doi: 10.1128/mbio.03696-21 PMC8787478 35073734

[ref12] Coggan K. A. Wolfgang M. C. (2012). Global regulatory pathways and cross-talk control Pseudomonas aeruginosa environmental lifestyle and virulence phenotype. Curr. Issues Mol. Biol. 14, 47–70. doi: 10.21775/cimb.014.047 22354680 PMC12747716

[ref13] Craig L. Pique M. E. Tainer J. A. (2004). Type IV pilus structure and bacterial pathogenicity. Nat. Rev. Microbiol. 2:363–378. doi: 10.1038/nrmicro885 15100690

[ref14] Dasgupta N. Ashare A. Hunninghake G. W. Yahr T. L. (2006). Transcriptional induction of the *Pseudomonas aeruginosa* type III secretion system by low Ca2+ and host cell contact proceeds through two distinct signaling pathways. Infect. Immun. 74, 3334–3341. doi: 10.1128/IAI.00090-06, PMID: 16714561 PMC1479281

[ref15] Dekimpe V. Déziel E. (2009). Revisiting the quorum-sensing hierarchy in Pseudomonas aeruginosa: the transcriptional regulator RhlR regulates LasR-specific factors. Microbiology 155, 712–723. doi: 10.1099/mic.0.022764-0, PMID: 19246742

[ref16] Dong Y.-H. Wang L.-H. Zhang L.-H. (2007). Quorum-quenching microbial infections: mechanisms and implications. Philos. Trans. R. Soc. B. Biol. Sci. 362, 1201–1211. doi: 10.1098/rstb.2007.2045, PMID: 17360274 PMC2435583

[ref17] Doss J. Culbertson K. Hahn D. Camacho J. Barekzi N. (2017). A review of phage therapy against bacterial pathogens of aquatic and terrestrial organisms. Viruses 9:50. doi: 10.3390/v9030050, PMID: 28335451 PMC5371805

[ref18] Drenkard E. Ausubel F. M. (2002). Pseudomonas biofilm formation and antibiotic resistance are linked to phenotypic variation. Nature 416, 740–743. doi: 10.1038/416740a, PMID: 11961556

[ref19] Dy R. L. Richter C. Salmond G. P. C. Fineran P. C. (2014). Remarkable mechanisms in microbes to resist phage infections. Ann. Rev. Virol. 1, 307–331. doi: 10.1146/annurev-virology-031413-085500, PMID: 26958724

[ref20] Ferrell E. Carty N. L. Colmer-Hamood J. A. Hamood A. N. West S. E. H. (2008). Regulation of *Pseudomonas aeruginosa* ptxR by Vfr. Microbiology 154, 431–439. doi: 10.1099/mic.0.2007/011577-0, PMID: 18227247

[ref21] Fineran P. C. Charpentier E. (2012). Memory of viral infections by CRISPR-Cas adaptive immune systems: acquisition of new information. Virology 434, 202–209. doi: 10.1016/j.virol.2012.10.003, PMID: 23123013

[ref22] Fuchs E. L. Brutinel E. D. Jones A. K. Fulcher N. B. Urbanowski M. L. Yahr T. L.. (2010). The Pseudomonas aeruginosa Vfr regulator controls global virulence factor expression through cyclic AMP-dependent and-independent mechanisms. J. Bacteriol. 192, 3553–3564. doi: 10.1128/JB.00363-10, PMID: 20494996 PMC2897347

[ref23] Fulcher N. B. Holliday P. M. Klem E. Cann M. J. Wolfgang M. C. (2010). The *Pseudomonas aeruginosa* Chp chemosensory system regulates intracellular cAMP levels by modulating adenylate cyclase activity. Mol. Microbiol. 76, 889–904. doi: 10.1111/j.1365-2958.2010.07135.x, PMID: 20345659 PMC2906755

[ref24] Harvey H. Bondy-Denomy J. Marquis H. Sztanko K. M. Davidson A. R. Burrows L. L. (2018). *Pseudomonas aeruginosa* defends against phages through type IV pilus glycosylation. Nat. Microbiol. 3, 47–52. doi: 10.1038/s41564-017-0061-y, PMID: 29133883

[ref25] Hoggarth A. Weaver A. Pu Q. Huang T. Schettler J. Chen F.. (2019). Mechanistic research holds promise for bacterial vaccines and phage therapies for *Pseudomonas aeruginosa*. Drug Des. Devel. Ther. 13, 909–924. doi: 10.2147/DDDT.S189847, PMID: 30936684 PMC6431001

[ref26] Høyland-Kroghsbo N. M. Muñoz K. A. Bassler B. L. (2018). Temperature, by controlling growth rate, regulates CRISPR-Cas activity in *Pseudomonas aeruginosa*. MBio 9, e02184–e02118. doi: 10.1128/mBio.02184-18 30425154 PMC6234860

[ref27] Høyland-Kroghsbo N. M. Paczkowski J. Mukherjee S. Broniewski J. Westra E. Bondy-Denomy J.. (2017). Quorum sensing controls the *Pseudomonas aeruginosa* CRISPR-Cas adaptive immune system. Proc. Natl. Acad. Sci. U. S. A 114, 131–135. doi: 10.1073/pnas.1617415113, PMID: 27849583 PMC5224376

[ref28] Ishimoto K. S. Lory S. (1989). Formation of pilin in Pseudomonas aeruginosa requires the alternative sigma factor (RpoN) of RNA polymerase. Proc. Natl. Acad. Sci. U. S. A. 86, 1954–1957. doi: 10.1073/pnas.86.6.1954, PMID: 2564676 PMC286823

[ref29] Jansen R. Embden J. D. A. van Gaastra W. Schouls L. M. (2002). Identification of genes that are associated with DNA repeats in prokaryotes. Mol. Microbiol. 43, 1565–1575. doi: 10.1046/j.1365-2958.2002.02839.x, PMID: 11952905

[ref30] Jones R. N. (2010). Microbial etiologies of hospital-acquired bacterial pneumonia and ventilator-associated bacterial pneumonia. Clin. Infect. Dis. 51, S81–S87. doi: 10.1086/653053, PMID: 20597676

[ref31] Kostylev M. Kim D. Y. Smalley N. E. Salukhe I. Greenberg E. P. Dandekar A. A. (2019). Evolution of the Pseudomonas aeruginosa quorum-sensing hierarchy. Proc. Natl. Acad. Sci. U.S.A. 116, 7027–7032. doi: 10.1073/pnas.1819796116, PMID: 30850547 PMC6452656

[ref32] Lansdown A. B. G. (2002). Calcium: a potential central regulator in wound healing in the skin. Wound Repair Regen. 10, 271–285. doi: 10.1046/j.1524-475X.2002.10502.x, PMID: 12406163

[ref33] Lee J. Zhang L. (2015). The hierarchy quorum sensing network in Pseudomonas aeruginosa. Protein Cell 6, 26–41. doi: 10.1007/s13238-014-0100-x, PMID: 25249263 PMC4286720

[ref34] Luo Y. Zhao K. Baker A. E. Kuchma S. L. Coggan K. A. Wolfgang M. C.. (2015). A hierarchical cascade of second messengers regulates *Pseudomonas aeruginosa* surface behaviors. MBio 6, e02456–e02414. doi: 10.1128/mBio.02456-14 25626906 PMC4324313

[ref35] Mah T.-F. Pitts B. Pellock B. Walker G. C. Stewart P. S. O’Toole G. A. (2003). A genetic basis for *Pseudomonas aeruginosa* biofilm antibiotic resistance. Nature 426, 306–310. doi: 10.1038/nature02122, PMID: 14628055

[ref36] Makarova K. S. Wolf Y. I. Alkhnbashi O. S. Costa F. Shah S. A. Saunders S. J.. (2015). An updated evolutionary classification of CRISPR–Cas systems. Nat. Rev. Microbiol. 13, 722–736. doi: 10.1038/nrmicro3569, PMID: 26411297 PMC5426118

[ref37] Mattick J. S. (2002). Type IV pili and twitching motility. Ann. Rev. Microbiol. 56, 289–314. doi: 10.1146/annurev.micro.56.012302.160938 12142488

[ref38] Miller J. H. (1972). “Assay of β-galactosidase”, in *Experiments In molecular genetics*. (Cold Spring Harbor: Cold Spring Harbor Laboratory Press), 352–355.

[ref39] Münch R. Hiller K. Barg H. Heldt D. Linz S. Wingender E.. (2003). PRODORIC: prokaryotic database of gene regulation. Nucleic Acids Res. 31, 266–269. doi: 10.1093/nar/gkg037, PMID: 12519998 PMC165484

[ref40] Passador L. Cook J. M. Gambello M. J. Rust L. Iglewski B. H. (1993). Expression of *Pseudomonas aeruginosa* virulence genes requires cell-to-cell communication. Science 260, 1127–1130. doi: 10.1126/science.8493556, PMID: 8493556

[ref41] Patterson A. G. Chang J. T. Taylor C. Fineran P. C. (2015). Regulation of the type IF CRISPR-Cas system by CRP-cAMP and GalM controls spacer acquisition and interference. Nucleic Acids Res. 43, 6038–6048. doi: 10.1093/nar/gkv517, PMID: 26007654 PMC4499141

[ref42] Perez-Rodriguez R. Haitjema C. Huang Q. Nam K. H. Bernardis S. Ke A.. (2011). Envelope stress is a trigger of CRISPR RNA-mediated DNA silencing in *Escherichia coli*. Mol. Microbiol. 79, 584–599. doi: 10.1111/j.1365-2958.2010.07482.x, PMID: 21255106 PMC3040579

[ref43] Saier M. H. Jr. Ramseier T. M. (1996). The catabolite repressor/activator (Cra) protein of enteric bacteria. J. Bacteriol. 178, 3411–3417. doi: 10.1128/jb.178.12.3411-3417.1996, PMID: 8655535 PMC178107

[ref44] Smith R. S. Wolfgang M. C. Lory S. (2004). An adenylate cyclase-controlled signaling network regulates *Pseudomonas aeruginosa* virulence in a mouse model of acute pneumonia. Infect. Immun. 72, 1677–1684. doi: 10.1128/IAI.72.3.1677-1684.2004, PMID: 14977975 PMC356001

[ref45] Stern A. Keren L. Wurtzel O. Amitai G. Sorek R. (2010). Self-targeting by CRISPR: gene regulation or autoimmunity? Trends Genet. 26, 335–340. doi: 10.1016/j.tig.2010.05.008, PMID: 20598393 PMC2910793

[ref46] Suh S.-J. Runyen-Janecky L. J. Maleniak T. C. Hager P. MacGregor C. H. Zielinski-Mozny N. A.. (2002). Effect of vfr mutation on global gene expression and catabolite repression control of *Pseudomonas aeruginosa*. Microbiology 148, 1561–1569. doi: 10.1099/00221287-148-5-1561, PMID: 11988531

[ref47] Taguchi F. Ichinose Y. (2011). Role of type IV pili in virulence of pseudomonas syringae pv. Tabaci 6605: correlation of motility, multidrug resistance, and HR-inducing activity on a non-host plant. Mol. Plant-Microbe Interact. 24, 1001–1011. doi: 10.1094/MPMI-02-11-0026, PMID: 21615203

[ref48] Topal H. Fulcher N. B. Bitterman J. Salazar E. Buck J. Levin L. R.. (2012). Crystal structure and regulation mechanisms of the CyaB adenylyl cyclase from the human pathogen *Pseudomonas aeruginosa*. J. Mol. Biol. 416, 271–286. doi: 10.1016/j.jmb.2011.12.045, PMID: 22226839 PMC3269522

[ref49] Vogan A. A. Higgs P. G. (2011). The advantages and disadvantages of horizontal gene transfer and the emergence of the first species. Biol. Direct 6:1. doi: 10.1186/1745-6150-6-1, PMID: 21199581 PMC3043529

[ref50] West S. E. Sample A. K. Runyen-Janecky L. J. (1994). The vfr gene product, required for *Pseudomonas aeruginosa* exotoxin a and protease production, belongs to the cyclic AMP receptor protein family. J. Bacteriol. 176, 7532–7542. doi: 10.1128/jb.176.24.7532-7542.1994, PMID: 8002577 PMC197210

[ref51] Westra E. R. Pul Ü. Heidrich N. Jore M. M. Lundgren M. Stratmann T.. (2010). H-NS-mediated repression of CRISPR-based immunity in *Escherichia coli* K12 can be relieved by the transcription activator LeuO. Mol. Microbiol. 77, 1380–1393. doi: 10.1111/j.1365-2958.2010.07315.x, PMID: 20659289

[ref52] Westra E. R. van Houte S. Oyesiku-Blakemore S. Makin B. Broniewski J. M. Best A.. (2015). Parasite exposure drives selective evolution of constitutive versus inducible defense. Curr. Biol. 25, 1043–1049. doi: 10.1016/j.cub.2015.01.065, PMID: 25772450

[ref53] Wiedenheft B. van Duijn E. Bultema J. B. Waghmare S. P. Zhou K. Barendregt A.. (2011). RNA-guided complex from a bacterial immune system enhances target recognition through seed sequence interactions. Proc. Natl. Acad. Sci. U. S. A. 108, 10092–10097. doi: 10.1073/pnas.1102716108, PMID: 21536913 PMC3121849

[ref54] Wolfgang M. C. Kulasekara B. R. Liang X. Boyd D. Wu K. Yang Q.. (2003a). Conservation of genome content and virulence determinants among clinical and environmental isolates of *Pseudomonas aeruginosa*. Proc. Natl. Acad. Sci. U. S. A. 100, 8484–8489. doi: 10.1073/pnas.0832438100, PMID: 12815109 PMC166255

[ref55] Wolfgang M. C. Lee V. T. Gilmore M. E. Lory S. (2003b). Coordinate regulation of bacterial virulence genes by a novel adenylate cyclase-dependent signaling pathway. Dev. Cell 4, 253–263. doi: 10.1016/S1534-5807(03)00019-4, PMID: 12586068

[ref56] Yahr T. L. Wolfgang M. C. (2006). Transcriptional regulation of the *Pseudomonas aeruginosa* type III secretion system. Mol. Microbiol. 62, 631–640. doi: 10.1111/j.1365-2958.2006.05412.x, PMID: 16995895

[ref57] Yang C. Chen Y. Huang H. Huang H. Tseng C. (2014). CRP represses the CRISPR/Cas system in *Escherichia coli*: evidence that endogenous CRISPR spacers impede phage P 1 replication. Mol. Microbiol. 92, 1072–1091. doi: 10.1111/mmi.12614, PMID: 24720807

