# Supplementary material for: The virulence factor regulator and quorum sensing regulate the type I-F CRISPR-Cas mediated horizontal gene transfer in Pseudomonas aeruginosa
Source: Front Microbiol. 2022 Sep 30;13:987656. doi: 10.3389/fmicb.2022.987656 (PMC9563714; doi:10.3389/fmicb.2022.987656)
Supplement: Supplementary file 1 [file Data_Sheet_1.PDF]

Supplementary data

Title: The Virulence factor regulator and Quorum Sensing regulate the Type I-F CRISPR-Cas mediated horizontal gene transfer in *Pseudomonas aeruginosa.*

Authors: Stephen Dela Ahator[1,3], Liu Yang[1,2], Wang Jianhe[1], Lian-Hui Zhang[1,*]

Affiliations:

[1] Guangdong Province Key Laboratory of Microbial Signals and Disease Control; Integrative Microbiology Research Center, South China Agricultural University, Guangzhou 510642, China

[2]Centro de Biotecnología y Genómica de Plantas, Universidad Politécnica de Madrid (UPM) – Instituto Nacional de Investigación y Tecnología Agraria y Alimentaria (INIA), Madrid, Spain

[3]Research group for Host Microbe Interactions, Department of Medical Biology, Faculty of Health Sciences, UiT The Arctic University of Norway, Tromsø, Norway

*__Correspondence email__: Lhzhang01@scau.edu.cn

Table S1: Strains and plasmids used in the study

STRAINS	DESCRIPTION	REFERENCE
E. coli		
**DH5α**	F– F80lacZDM15 endA1hsdR17 (rk – mk –) supE44 thi -1 gyrA96 D(lacZYA-argF)	Lab collection
**S17-1 λ*PIR***	*recA pro (RP4-2Tet::Mu Kan::Tn7)*	Lab collection
**BL21**	F– ompT hsdSB (rB-mB -) gal dcm rne131 (DE3)	Invitrogen
P. aeruginosa		
**UCBPP_PA14**	Wild type strain	Lab collection
Δvfr	vfr in-frame deletion mutant	This study
Δcas3	*Cas3* in-frame deletion mutant	This study
ΔlasIΔrhlI	*AHL* null mutant	This study
ΔcyaB	*cyaB* in-frame deletion mutant	This study
ΔvfrΔlasΔrhlI	In-frame deletion mutant of *lasI, rhlI* and *vfr*	This study
**Plasmids**		
**pK18mobsacB**	Broad-host-range gene replacement suicide vector, sacB, Gm^r	Lab collection
**pBT20**	Mariner transposon mutagenesis vector, Gm^R	Lab collection
**pUCP19**	*E. coli – P. aeruginosa* shuttle vector with lac promoter (plac), Amp^R/Cb^R	Lab collection
**pUCPT**	*E. coli – P. aeruginosa* shuttle vector with lac promoter (plac), oriT, Amp^R/Cb^R	Lab collection
**pET28b**	Protein expression vector, Amp^R	Lab collection
**pME2-lacZ**	pME6010 carrying a full-length lacZ, Tc^R	Lab collection
**pMEPcas1-lacZ**	pME2-lacZ carrying *cas1* promoter fused to lacZ.	This study
**pKCPc1z**	pK18mobsacB carrying intact *cas1* promoter lacZ fusion for chromosomal integration	This study
**pKCs1VBSlz**	pK18mobsacB carrying altered Vfr binding site in the *cas1* promoter fused to lacZ fusion for chromosomal integration	This study
**pUCPTSp1**	pUCPT carrying CRISPR2 Spacer1	(Cady et al., 2012)(HYLAND-KROGHSBO, 2017)
**pUCPTSp2n**	pUCPT carrying CRISPR2 Spacer1 with 2 nucleotide substitutions after the GG PAM sequence	This study
**pUCPTSp4n**	pUCPT carrying CRISPR2 Spacer1 with 4 nucleotide substitutions after the GG PAM sequence	This study

Table S2: Oligonucleotides used in the study

Primer name	PRIMER SEQUENCE (5'-3')	Notes
**IN-FRAME DELETION PRIMERS**		
**vfrUpF**	CTATGACATGATTACGAATTCTGACCTGGGCCTCCTTGAGAC	*vfr* up deletion fragment
**vfrUpR**	CGATCTCCTGGCGGGTGATCTCGCCGGCATAGATGATGGTGC	
**vfrDnF**	CGCCGGCATAGATGATGGTGCAGATCACCCGCCAGGAGATCG	*vfr* down deletion fragment
**vfrDnR**	CGACGGCCAGTGCCAAGCTTTTCCAGGGCGCCGATGCCTAT	
**lasIUpF**	CTATGACATGATTACGAATTCGGTGCCGGACTGGCCTTCGAA	*lasI* up deletion fragment
**lasIUpR**	CTTCAGGTGCGGACCGAAGCG ACGTCCCAGCCTTTGCGCTCC	
**lasIDnF**	ACGTCCCAGCCTTTGCGCTCCCGCTTCGGTCCGCACCTGAAG	*lasI* down deletion fragment
**lasIDnR**	CGACGGCCAGTGCCAAGCTTGTCCGCAATGCGCAGGCGTTC	
**rhlUpF**	CTATGACATGATTACGAATTCCGGAAATGGTGGTCTGGAGC	*rhlI* up deletion fragment
**rhlUpR**	CGAAACGGCTGACGACCTCACATGACCAAGTCCCCGTGTC	
**rhlDnF**	CATGACCAAGTCCCCGTGTCTGAGGTCGTCAGCCGTTTCG	*rhlI* down deletion fragment
**rhlDnR**	CGACGGCCAGTGCCAAGCTTGGCGAACTTCTCGTTGGTGC	
**cyaBUpF**	CTATGACATGATTACGAATTCGCCGAGTTCTACCCCTACTAC	*cyaB up* deletion fragment
**cyaBUpR**	GGATGACCCGTTCCTTGTCGTATACTGGGTGTAGGTTCCCGC	
**cyaBDnF**	ATACTGGGTGTAGGTTCCCGCACGACAAGGAACGGGTCATCC	*cyaB down* deletion fragment
**cyaBDnR**	CGACGGCCAGTGCCAAGCTTGCGTTCCTCGATCTCGAACTG	
**cas3upF**	CTATGACATGATTACGAATTCCGTCGATGCCACCGCACTGGC	*Cas3* up deletion fragment
**cas3upR**	GTCGTCCTCGTCCGGCAGCAGGGCACCGCACCCTGGGCGTTG	*Cas3* deletion
**cas3dnF**	GGCACCGCACCCTGGGCGTTGCTGCTGCCGGACGAGGACGAC	*Cas3* down deletion fragment
**cas3dnR**	CGACGGCCAGTGCCAAGCTTACGCGGGAGCGCCTTTGACGG	
**COMPLEMENTATION**		
**VfrUp**	GACCATGATTACGCCAAGCTTGGCATGGTAGCTATTACCCAC	*vfr* complementation
**VfrDn**	AAAACGACGGCCAGTGAATTCTCAGCGGGTGCCGAAGACCAC	
**cyaBUp**	GACCATGATTACGCCAAGCTTATGAAGCCTACCCTCCCCGAC	*cyaB* complementation
**cyaBDn**	AAAACGACGGCCAGTGAATTCTCAGAGGATGACCTTGTCGCG	
**TRANSPOSON MUTAGENESIS (TAIL COLONY PCR)**		
**Ad3**	GTCGASWGANAWGNA	All three tails
**Gt447**	GTGCAAGCAGATTACGGTGACGAT	Tail 1
**Gt464**	TGACGATCCCGCAGTGGCTCTC	Tail 2
**Gt487**	ATACAAAGTTGGGCATACG	Tail 3
**Gt531**	GACCCAAGTACCGCCACC	Sequencing
**QUANTITATIVE REAL TIME PCR**		
**Cas1up**	CGGGGTATTGGTAGGCTTCT	*Cas1* expression
**Cas1dn**	TCGACGTCCACTTCGTTG	*Cas1* expression
**Cas3up**	CTGCACAGTCGCAAGCTC	Cas3 expression
**Cas3dn**	GCAGTCGTAGGCCTTGTCC	Cas3 expression

**Csy1up**	AAATCGCTTCGCCAACTG	*Csy1* expression
**Csy1dn**	CGGGCTACCCAGTATCTGAC	*Csy1* expression
**csy2Up**	TTCAACCTGACCCGCAAT	*Csy2* expression
**csy2Dn**	CGAGCAGCAGACTGACCTC	*Csy2* expression
**csy3Up**	ATCCCAGGGCAAAGCCTAT	*Csy3* expression
**csy3Dn**	TCGAGCAGCGTATAGAAGTCC	*Csy3* expression
**Csy4up**	TCAGGCGAAAAGCAATCC	*Csy4* expression
**Csy4dn**	CCTCCTCCTCACTCAGATCG	*Csy4* expression
**ProC Up**	TTCGAAGCACTAGTGGAGCA	Endogenous control
**ProC Dn**	TTATTGGCCAAGCTGTTCG	Endogenous control
**EMSA PRIMERS**		
**PlasR Up**	GCGCTACGTTCTTCTTAAACT	*lasR* promoter
**PlasR Dn**	CGCCGCGAGGTCGCCCCCTTG	
**PrhlR Up**	ACGGTGCTGGCATAACAG	*rhlR* promoter
**PrhlR Dn**	TGCAGTAAGCCCTGATCG	
**Pcas1Up**	AACAGCCGCCGGGTTCGCTGC	Promoter of *cas1*
**Pcas1Dn**	GTTGACCGTGGCCTGTCCCTA	
**Pcas1 VBSUpR**	CTGGGGATGGAGCGCATATAAGCAAGCGAGGCACATTGTCGTG	*cas1* promoter with altered Vfr binding site
**Pcas1 VBSDnF**	GAAATATGCGCTCCATCCCCAGACGAAAACGGGGTAGCGGCAA	
**PcyaB Up**	CATGCGCTGGAGAGGATCCCT	*cyaB* promoter
**PcyaB Dn**	TCTGACCCATCCGCGCGACTG	
**PptxR Up**	CCTGTCATTCCTTAGCCGGGA	*ptxR* promoter
**PptxR Dn**	GGACCTGGTACCTCTTGGTGC	
**CHROMOSOMAL LACZ INTEGRATION**		
**Cs1upf**	CTATGACATGATTACGAATTCTCGAACAGCCCGGGTTCGGTC	Chromosomal Integrative P*cas1-lacZ* fusion construct
**Cs1upr**	CCAGTGAATCCGTAATCATGGTCATCATGTTGACCGTGGCCTGTCC	
**Cs1lzupf**	GGACAGGCCACGGTCAACATGATGACCATGATTACGGATTCACTGG	
**Cs1lzdnr**	TTCGCTGGGAGAAATGTCGTCTTATTTTTGACACCAGACCAACTGG	
**Cs1dnf**	CCAGTTGGTCTGGTGTCAAAAATAAGACGACATTTCTCCCAGCGAA	
**Cs1dnr**	CGACGGCCAGTGCCAAGCTTCTTCCACGGCAACTGCCAGTG	
**Cs1VBSUpR**	CTGGGGATGGAGCGCATATTTCCAAGCGAGGCACATTGTCGTG	Integrative P*cas1-lacZ* with altered VBS
**Cs1VBSDnF**	GAAATATGCGCTCCATCCCCAGACGAAAACGGGGTAGCGGCAA	
**SPACER ACQUISITION**		
**Cr2sp1up**	TTGACGAACGCCGTCCAGAAG	CRISPR2 expansion
**Cr2sp1dn**	GAGGGTTTCTGGCGGGAA	
**CRISPR-Cas targeted oligonucleotides**		
**pUCPTSp1**	ACCGCGCTCGACTACTACAACGTCCGGCTGAT**GG**	CRISPR2 spacer 1

**pUCPTSp2n**	ACCGCGCTCGACTACTACAACGTCCGGCTGTC**GG**	2nucleotide mutations
**pUCPTSp4n**	ACCGCGCTCGACTACTACAACGTCCGGCGACC**GG**	4 nucleotide mutations
**VFR-HIS PROTEIN PRIMERS**		
**VfR-HisUp**	TAAGAAGGAGATATACCATGGATGTCGGAACAACTGTCTACC	Vfr-His tagged protein expression
**VfR-HisDn**	TGGTGGTGCTCGAGTGCGGCCGC TCAGCGACGGGCGCTGAGATG	

R=reverse, F= forward and Dn= down.

Supplementary figure 1

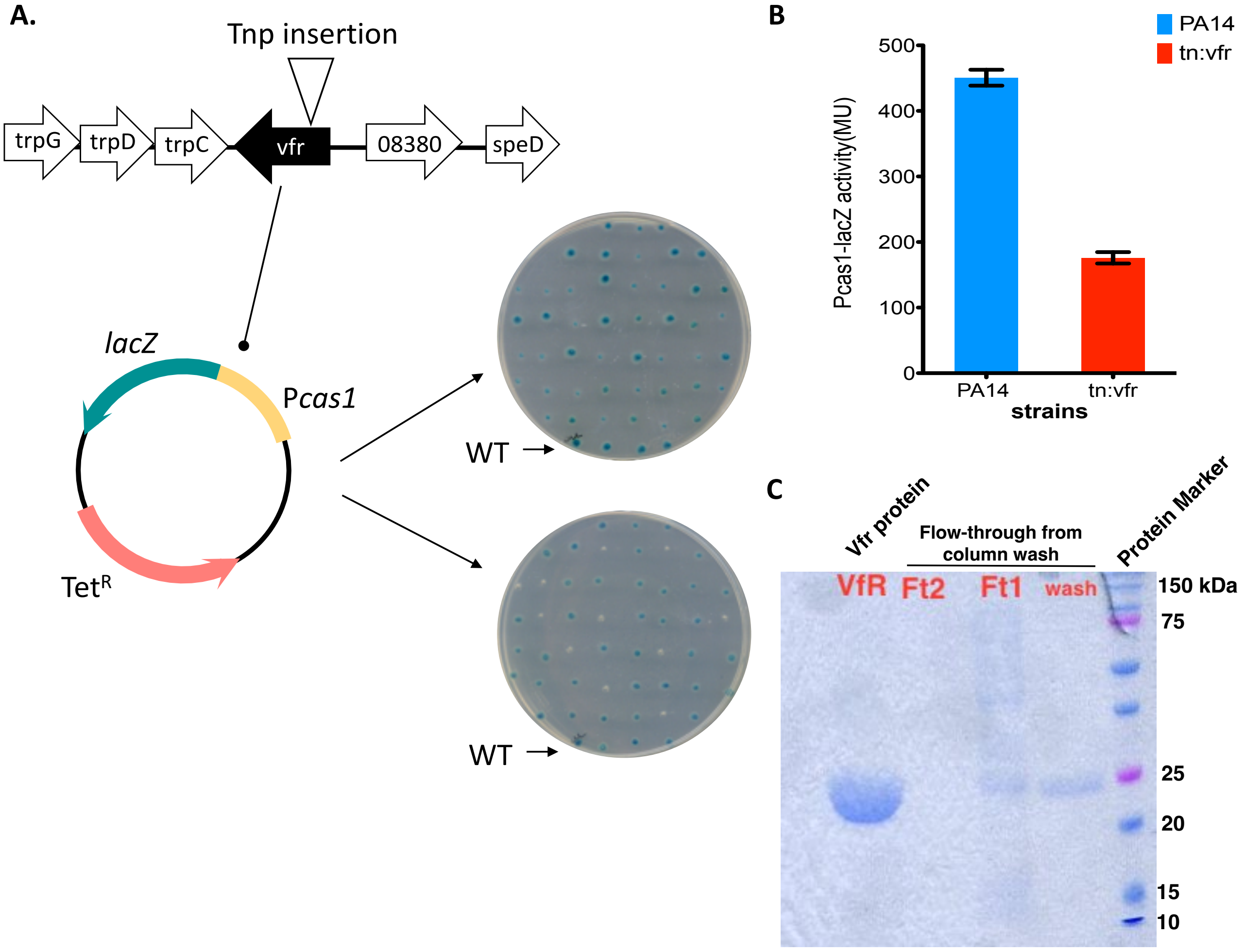

(**A**) Schematic of random transposon mutagenesis in *P. aeruginosa*. Random transposon mutagenesis was performed using the mariner transposon, pBT20 in the WT PA14 carrying the construct pME-*Pcas1-lacZ*. In all more than 5000 colonies were screened. Visual inspection was performed by comparing blue coloration of transposon mutants with the parental mating strain on the selection plate containing X-gal. Transposon inserts that disrupt positive regulators of CRISPR-Cas resulted in lighter blue colonies compared to the wild type (WT). White colonies were ignored as they signified insertion into the plasmid. (**B)**. β-galactosidase assay for the *Pcas1-lacZ* activity in wild type PA14 and *vfr* transposon mutant (tn:*vfR*). (**C**). SDS_PAGE image showing the 24 kDa VfR protein expressed from the pET28b vector in *E. coli* BL21.

Supplementary figure 2

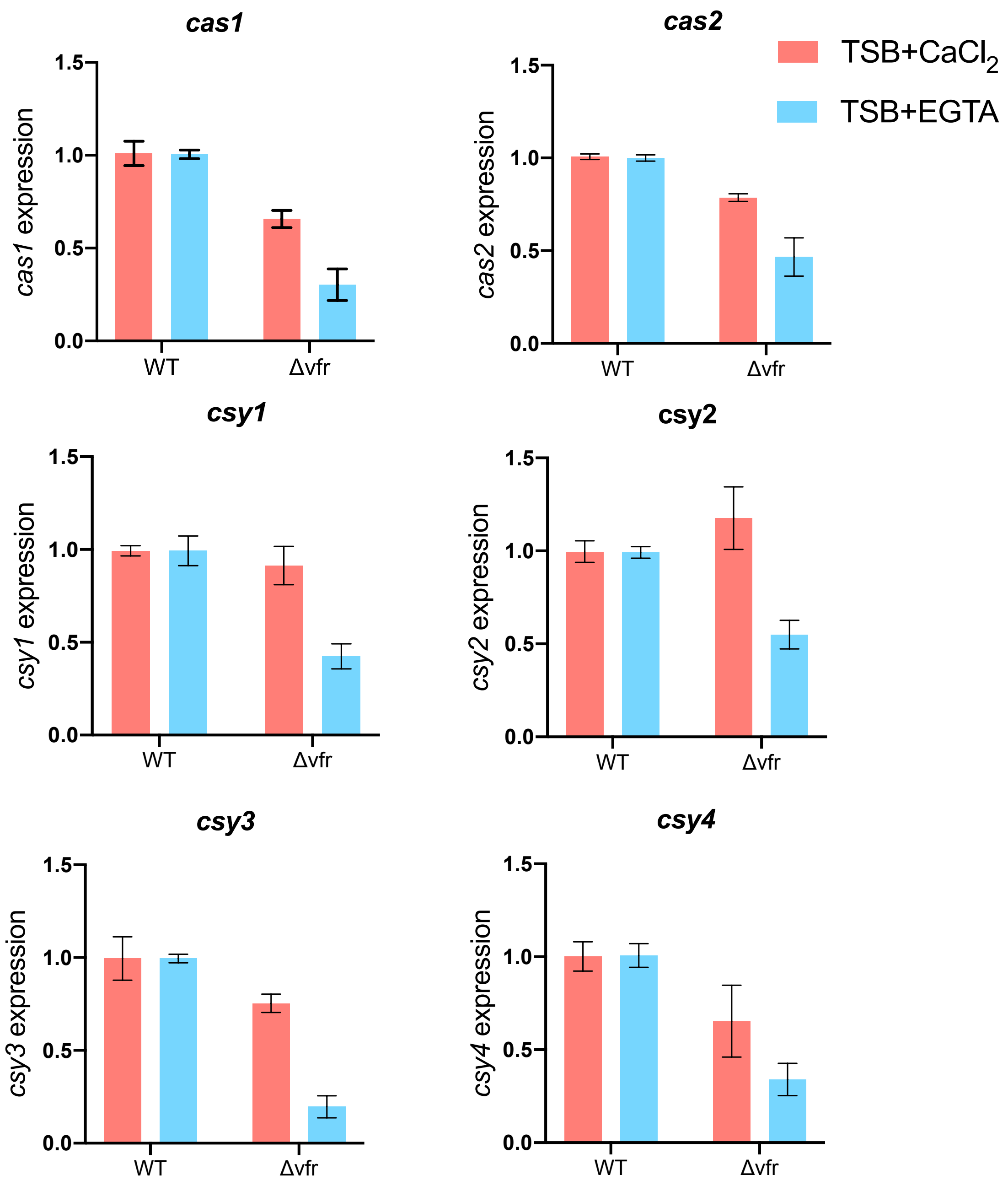

Fig S2**. Vfr regulates the expression of cas genes.**

Quantitative real-time PCR analysis of cas genes: *cas1,cas2,csy1,csy2,csy3,* and *csy4* in the Δ*vfr* strains and wild type PA14 grown in TSB+$CaCl_2$ and TSB+EGTA. The mean expression levels of the cas genes in the WT are normalized to 1. Data shown are the mean with SD from n = 4 replicates.

Supplementary figure 3

aacagccgccgggttcgctgccgtctaggcagaaccaccctccccatccca

ctaccaaacatccgaatataaagttcctaccccgcccgccagcctcgcccgt

LasR/RhlR box

ccacgacaatgtgcctcgcttggaagctcacgctcctcacacagacgaaaa

Vfr CBS LasR/RhlR box

cggggtagcggcaatcggccatatccgctaaacagttgccttggcg

cagaattcgatagatccgatagggacaggccacggtcaac ATG

Figure S3
DNA sequence of *cas1* promoter region. Underlined sequence represents the Vfr binding site. The two *lasR/rhlR* boxes are labelled in bold red and shaded. The Vfr binding site partially overlaps with one of the *lasR/rhlR* binding site. The binding sites of the Vfr and the AHL QS regulators were identified using the prodoric database.

Supplementary figure 4

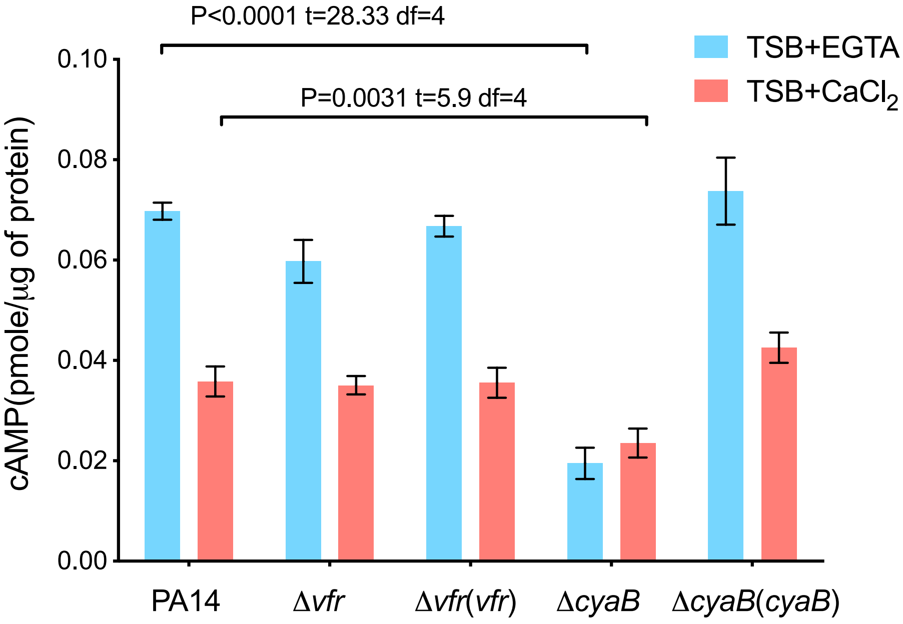

Fig S4

Intracellular cAMP concentration of Δ*vfr* and Δ*cyaB* with their respective complemented strains Δ*vfr(vfr)* and Δ*cyaB(cyaB)* and the wild type PA14. Strains were grown in calcium depleted and calcium rich media. The concentration of intracellular cAMP is expressed as pmole per μg of total cellular protein. Data represents the mean± SD of 4 independent repeats. Statistical analysis was calculated using student t-test (P≤ 0.05 is considered significant).

Supplementary figure 5

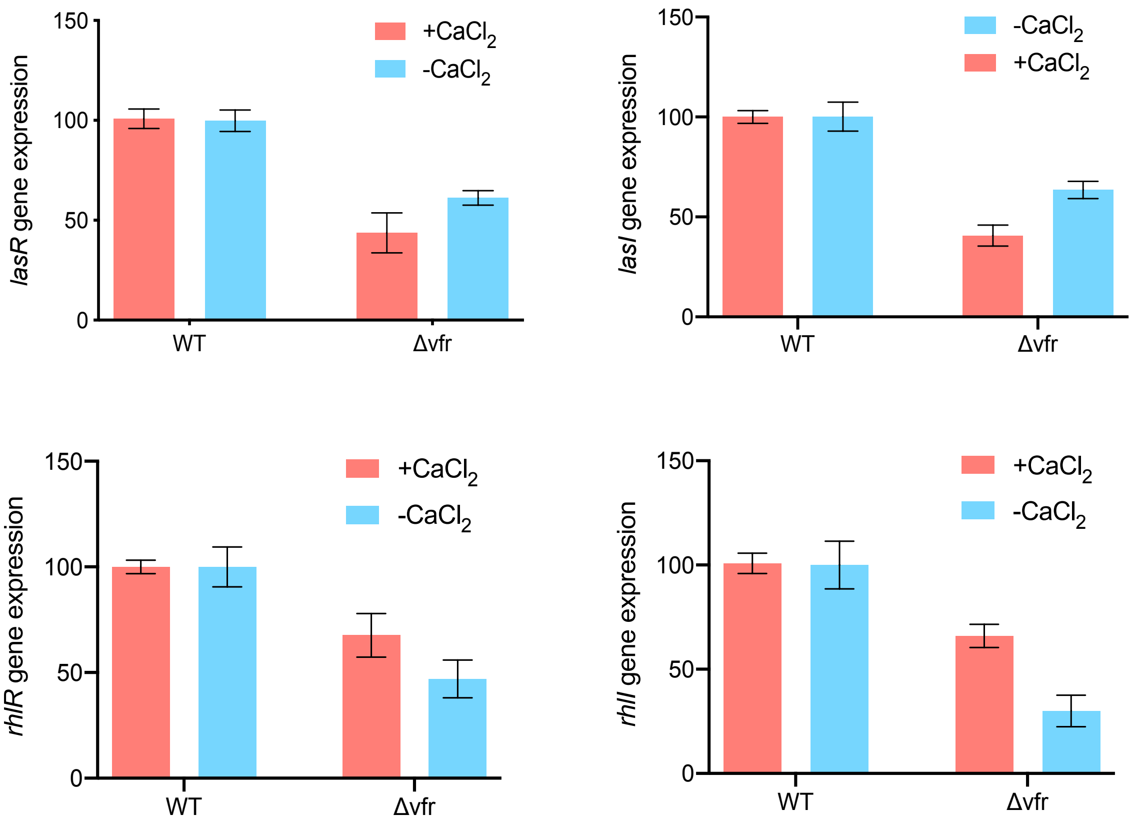

Fig S5**. Vfr regulates the CRISPR-Cas system via AHL QS.**
Quantitative real-time PCR analysis of AHL-QS genes *lasI, lasR, rhlI and rhlR* in the Δ*vfr* strains and wild type grown in TSB+$CaCl_2$ and TSB+EGTA. The mean expression levels of the QS genes in the WT are normalized to 1. Data shown are the mean with SD from n = 4 replicates.

Supplementary figure 6.

A

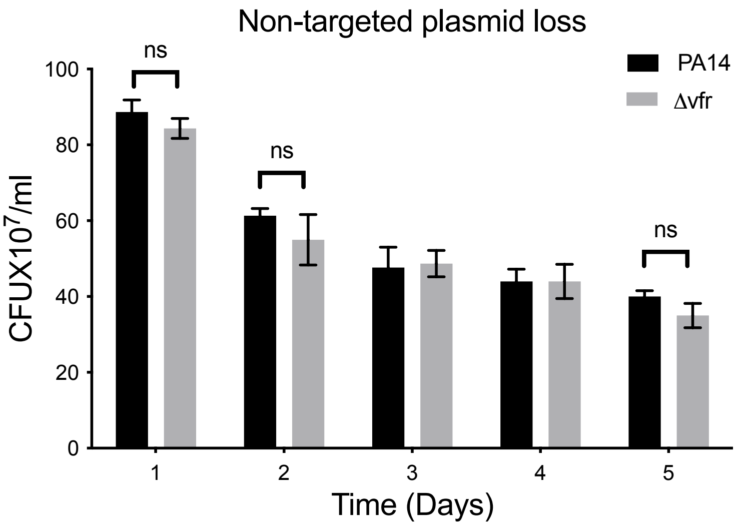

B

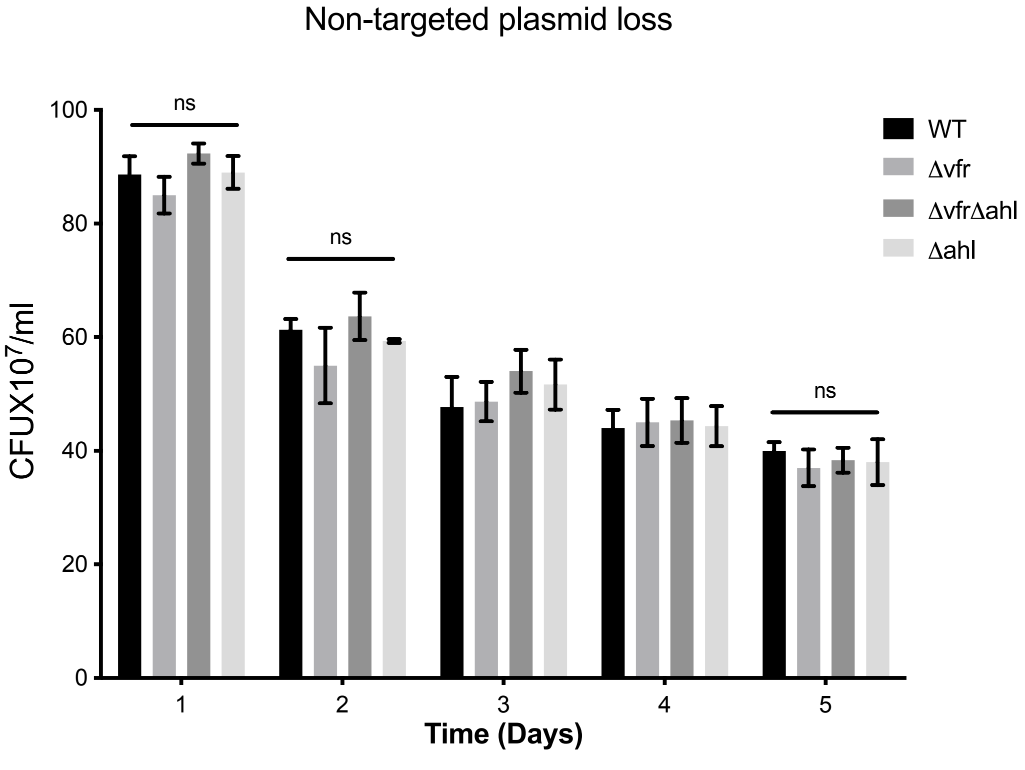

Figure S6.

Retention of the CRISPR-Cas non-targeted plasmid in *P. aeruginosa* strains passaged in calcium depleted media (TSB+EGTA)(**A**) and calcium rich media (TSB+ $CaCl_2$)(**B**) over 5 days. The plasmid loss was scored by counting positive colonies on plates containing carbenicillin. The data represent the mean ± SD (n = 6). Statistical significance was calculated using student's t-test and Bonferroni-Dunn method multiple comparison test (P value <0.05 is considered significant).

Figure S7. Model of CRISPR-Cas system cas regulation in *P. aeruginosa*.

A

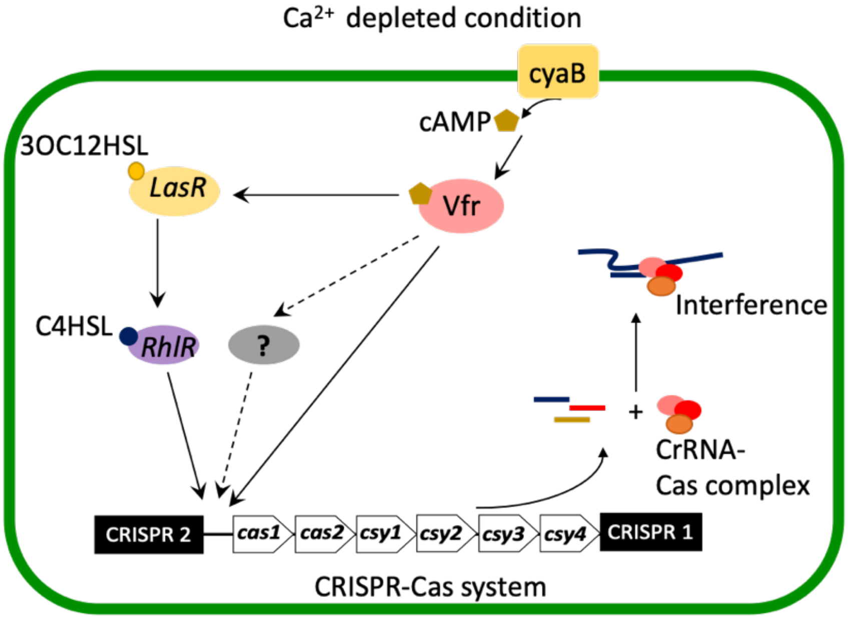

B

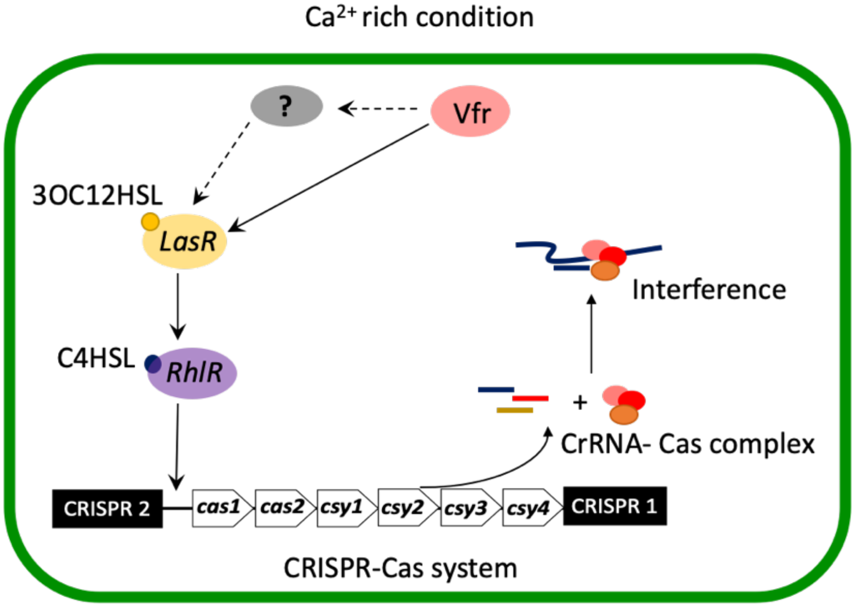

Vfr can regulate the CRISPR-Cas system via the AHL QS system under both calcium-rich and calcium calcium-depleted conditions. Also, Vfr can directly regulate the CRISPR-Cas system in a cAMP dependent manner under calcium-depleted condition. Due to the global regulon of Vfr, it is possible for alternative factors to mediate its regulation of the CRISPR-Cas system under both conditions tested, which may account for the inability of exogenous AHL to fully rescue the expression of the cas gene in the Δ*vfr*Δ*lasI*Δ*rhlI* mutant. The Vfr-QS-CRISPR-Cas cascade controls CRISPR-mediated interference and adaptation. Dashed straight arrows represent undetermined alternative pathways. Solid arrows represent experimentally determined pathways. Curved arrows represent derived products.

Reference:

Cady, K. C., Bondy-Denomy, J., Heussler, G. E., Davidson, A. R., & O'Toole, G. A. (2012). The CRISPR/Cas adaptive immune system of Pseudomonas aeruginosa mediates resistance to naturally occurring and engineered phages. *Journal of Bacteriology*, *194*(21), 5728–5738.

Høyland-Kroghsbo, N. M., Paczkowski, J., Mukherjee, S., Broniewski, J., Westra, E., Bondy-Denomy, J., & Bassler, B. L. (2017). Quorum sensing controls the Pseudomonas aeruginosa CRISPR-Cas adaptive immune system. *Proceedings of the National Academy of Sciences*, *114*(1), 131–135. https://doi.org/10.1073/pnas.1617415113
